# Identification of Novel Ribonucleotide Reductase Inhibitors for Therapeutic Application in Bile Tract Cancer: An Advanced Pharmacoinformatics Study

**DOI:** 10.3390/biom12091279

**Published:** 2022-09-10

**Authors:** Md Ataul Islam, Mayuri Makarand Barshetty, Sridhar Srinivasan, Dawood Babu Dudekula, V. P. Subramanyam Rallabandi, Sameer Mohammed, Sathishkumar Natarajan, Junhyung Park

**Affiliations:** 1 3BIGS Omicscore Private Limited, 909 Lavelle Building, Richmond Circle, Bangalore 560025, India; 2 3BIGS Co., Ltd., B-831, Geumgang Penterium IX Tower, Hwaseong 18469, Korea

**Keywords:** biliary tract cancer, human ribonucleotide reductase, de novo design, molecular docking, molecular dynamics simulation

## Abstract

Biliary tract cancer (BTC) is constituted by a heterogeneous group of malignant tumors that may develop in the biliary tract, and it is the second most common liver cancer. Human ribonucleotide reductase M1 (hRRM1) has already been proven to be a potential BTC target. In the current study, a de novo design approach was used to generate novel and effective chemical therapeutics for BTC. A set of comprehensive pharmacoinformatics approaches was implemented and, finally, seventeen potential molecules were found to be effective for the modulation of hRRM1 activity. Molecular docking, negative image-based ShaEP scoring, absolute binding free energy, in silico pharmacokinetics, and toxicity assessments corroborated the potentiality of the selected molecules. Almost all molecules showed higher affinity in comparison to gemcitabine and naphthyl salicylic acyl hydrazone (NSAH). On binding interaction analysis, a number of critical amino acids was found to hold the molecules at the active site cavity. The molecular dynamics (MD) simulation study also indicated the stability between protein and ligands. High negative MM-GBSA (molecular mechanics generalized Born and surface area) binding free energy indicated the potentiality of the molecules. Therefore, the proposed molecules might have the potential to be effective therapeutics for the management of BTC.

## 1. Introduction

Cholangiocarcinoma or biliary tract cancer (BTC) is the highly aggressive and second most common liver cancer worldwide followed by hepatocellular carcinoma (HCC) [1]. The BTC originated from the biliary epithelium of the small ducts in the periphery of the liver and the main ducts of the hilum. The BTC is covering about less than one percent of all human cancers and about 15% of primary liver cancer [1]. Every year, more than 8000 people are diagnosed with BTC in the United States of America (USA). In particular, BTC is found to be diffident in the western world, having an incidence of about 2 persons per 100,000 in each year. It is a matter of concern that the part of Southern Asia including China, Thailand, and Korea are more prone to have BTC evidence of a much higher rate in comparison to Europe and the USA. In particular, BTC depends upon the demographic distribution of the risk factors and most probably depends upon the ethnic race. It is evident that about 0.1 per 100,000 is found in Australia, and 110 out of 100,000 are found in Thailand. The data of the last several decades suggest the steady rise of BTC all over the world [2,3,4,5,6,7,8]. It is important to note that the quiet presence of BTC and its highly insistent nature influence the mortality rates globally [9]. The surgical resection is the only effective curative therapy for the BTC, but it is asymptotic in nature at the beginning and generally diagnosed at an advanced stage that leads to a compromise of treatment options [10,11]. A large-scale sharing of awareness and knowledge along with diagnosis and therapies are gradually improving but no significant prognosis has been improved in the last decade, which is substantiated by the five-year survival of 7–20% [12,13,14,15,16,17,18,19].

Ribonucleotide reductase (RR) is one of the effective and potential therapeutic cancer targets and important to regulate the enzyme in the DNA synthesis and repair pathway. It is unique and plays an important role to reduce the nucleotide diphosphates (NDPs) to 2′ deoxyribonucleotide diphosphates (dNDPs). Further, in the rate-limiting step, deoxyribonucleotide triphosphates (dNTPs) are synthesized from purine- and cytidine-based DNA precursors [20] and maintain the balanced pools of it in the cell [21]. In the replication and repair of DNA in all living cells, the RR is essential to control cell proliferation and maintain genome stability [22,23]. Lack of continuous proper concentrations of dNTPs may be harmful to the cells and may lead to DNA breakage, mutagenesis, and cell death [24]. In the stage of cancer progression, deficiency of dNTPs may be observed due to uncontrolled cell proliferation, which further leads to replication stress followed by enhancement of genomic instability [25,26]. On the other hand, a high concentration of dNTP is responsible for mutagenesis development [27,28].

Structurally, RR is a heterodimeric compound comprised of two subunits, RR1 (α) and RR2 (ß). RR1 is mainly responsible for the activation of several oncogenes [29], whereas RR2 is overexpressed in the related increased Raf-1 membrane-associated protein and mitogen-activated kinase (MAPK) activity [30] and resistant to cytotoxic therapy [31]. In particular, RR1 is the catalytic subunit that possesses the catalytic site (C-site), two allosteric sites, the specificity site (S-site), and the activity site (A site). The RR2 subunit houses a free radical essential for catalysis [32]. The presence of three cysteine residues in the catalytic site conducts the thiol-based redox chemistry in the reduction of ribose substrate to 2′-deoxyriobose [32]. RR1, i.e., α subunit, is a dimer form in the presence of the allosteric effectors dATP and ATP in the form of α_6_ß_2_ and α_6_ß_i_ (where i = 2, 4, and 6), respectively [33]. The human RR (hRR) is a crucial target for cancer therapy and consists of two subunits, namely, hRRM1 and hRRM2 [34]. It is evident that nucleoside analogs such as gemcitabine, clofarabine, and cladribine nucleotides inhibit hRRM1 by stabilizing a form of the α_6_ complex [35,36,37,38]. The hRRM2 subunit is the non-heme iron and a tyrosine-free radical that is essential for the enzymatic reduction of ribonucleotides [39]. The inhibition of hRRM1 by a number of effective anti-cancer hRR nucleotide-analog inhibitors including gemcitabine may trigger the cancer cells to ionizing radiation and to DNA-damaging drugs [40]. Beyond the above, the capability of inhibition of hRR by the anti-cancer analog hRR decreases dNTPs, which enhances the ability of gemcitabine triphosphate to be incorporated into growing DNA strands by DNA polymerase. Gemcitabine is an extensively effective and potential chemical entity for the treatment and management of BTC [41,42]. Several studies have already provided sufficient evidence in favor of gemcitabine alone as effective against BTC up to 30% [43,44,45]. Moreover, along with capecitabine or platinum analogs, gemcitabine produced response rates of 26 to 53% in clinical trial phase II [46,47]. The combination of gemcitabine and cisplatin has been tested for BTC in several clinical trial studies and was found to be about 21 to 53% effective [48]. A number of studies already reported the serious adverse effect of gemcitabine on its cytotoxicity to the normal cells that lead to termination of DNA chains and irreversible inhibition of hRR [49,50,51]. Therefore, targeting the hMMR1 subunit of the hRR to design and identification of promising chemical entities will be an effective approach to treat and manage the BTC.

Identification of any chemical component for the treatment of a certain disease is a critically complex, challenging, extensively time-consuming, and high-cost-intensive approach in the pharmaceutical industry. It has been reported that bringing a single drug molecule into the market starting from basic research may take about 12–16 years of time and a cost estimated at USD 2.5 million [52,53,54,55]. Recently, it has been reported that only one successful compound may be found from about 10,000 molecules through research and development [56,57]. One of the important aims of the European Union Sustainable Development Goals is to provide good health and wellbeing for everyone, and drugs should be available to the common people at an affordable cost [58]. Hence, the current drug development protocols must be changed to make it economically sustainable and feasible to the research communities. The use of advanced computational resources and power in the pharmaceutical industry enhanced the drug discovery pipeline with lower cost and minimum time along with trivial animal sacrifice. Virtual screening of any existing database or computationally designed novel compounds through de novo design has already gained exciting popularity in the scientific communities across the globe. De novo drug design (DNDD) is the method to design novel chemical compounds with the help of computational approaches without any prior relationships [59]. The meaning of 'de novo' is 'from the beginning', which indicates that novel molecules can be designed without any pre-defined template(s) [60]. In this method, one can explore the boundless chemical space and design molecules that have intellectual properties that are effective for novel therapies [59]. Applications of several computational strategies including negative image-based (NIB) screening [61], molecular docking [62], un-supervised pharmacophoric features assessment [63], in silico pharmacokinetics and toxicity [64], PLANTS-based docking [65], absolute binding free energy assessment [66], and molecular dynamics (MD) simulation [67] to reduce the chemical space have already proven their efficiency and efficacy in drug discovery research. NIB screening can directly be used to develop the models from the receptor cavity of the macromolecule using the shape and electrostatic complementary between active site and bound small molecule. Molecular docking is the pivotal methodology for screening of small to ultra large chemical databases for any specific drug target. Pharmacokinetic and toxicity assessment of chemical compounds have become pioneer approaches to explore the promising chemical entities. MD simulation followed by binding affinity calculation have been widely adopted by the scientific community to explore the dynamic behavior of the small molecules inside the receptor cavity of the macromolecule. Hence, to identify promising molecules for the effective therapeutic application in BTC, DNDD-based design of hRRM1 inhibitors followed by screening through the above computational methodologies was the main objective of the current work. The credential of the work is supported by the identification of several promising molecules and their strong affinity to being potential hRRM1 inhibitors subjected to experimental validation.

## 2. Materials and Methods

The DNDD approach was used to design a set of novel compounds for the hRRM1, and subsequently, several screening parameters were implemented to reduce the chemical space. In this pipeline, a rigorous approach such as MD simulation followed by molecular mechanics generalized Born and surface area (MM-GBSA)-based binding free energy calculation were also performed to screen out the inactive molecules. For the comparative analyses, two standard molecules, namely, gemcitabine and naphthyl salicylic acyl hydrazone (NSAH), were used throughout the study. Gemcitabine has already been approved as a potential drug molecule for the treatment of advanced BTC [68,69]. In a recent study, NSAH was reported as a unique potential hRRM1 inhibitor [70].

### 2.1. Protein Target Selection and Preparation

The crystal structure of hRRM1 was obtained from the Research Collaboratory for Structural Bioinformatics (RCSB) Protein Data Bank (PDB), California, USA [71]. The PDB is the largest resource of experimental three-dimensional (3D) structures of macromolecules. The 3D coordinates of hRRM1 for the current experiment were considered to have PDB ID: 3HND [33]. The resolution and R-value of the structure were found to be 3.21 Å and 0.254, respectively. The sequence length of the protein was found to be 792 amino acids long. The missing atoms and amino acids were repaired using the online server CHARMM-GUI [72]. The CHARMM-GUI was developed and is maintained by Dr. Im’s research group at Lehigh University, Bethlehem, USA. AutoDock Tools (ADT) [73] was used to prepare the molecule before it was used in any in silico study. The missing atoms and amino acids were checked and repaired. Co-crystal water molecules and all hetero atoms were removed. The hydrogens and Gasteiger charges were added. The prepared protein molecule was saved in .pdb format for further use. Prior to using the protein molecule in AutoDock Vina (ADV) [74], the prepared protein was assigned the AD4 (AutoDock4) atom type and saved in the .pdbqt file format. Both ADT and ADV are maintained by The Scripps Research Institute, LaJolla, CA, USA.

### 2.2. De Novo Design Using LigBuilder

DNDD is one of the pivotal drug discovery approaches to design and identify novel molecules for any specific target. In order to design novel chemical entities targeting hRRM1, the open source LigBuilder v3.0, Perking University, Beijing, China [75] was used, which is an extensively and highly acknowledged de novo molecule generation tool. This tool was developed by the Institute of Physical Chemistry, Peking University, China. It is a genetic algorithm-based tool to construct the ligands library. More precisely, the LigBuilder develops the pool of small molecules according to the user-defined active site of the target molecule, and, subsequently, optimization and screening are done based on the input 3D structure of the protein molecule. The LigBuilder executes with two modules, namely, ‘cavity’ and ‘build.’ To detect the cavities in a protein molecule, the ‘cavity’ module considers the protein 3D structure and best docked/co-crystal ligand in .mol2 format. The best-docked pose of gemcitabine in .mol2 and 3D coordinates of hRRM1 were given for the ‘cavity’ module to explore the binding sites and generate the required data for the ‘build’ module. In LigBuilder, ‘build’ is a core functional module for the design and subsequent analysis of the molecules obtained based on ‘cavity’ output data. Using the fragment-based design methodology, the ‘build’ module mainly produces a set of molecules according to the input parameters. Along with binding site data derived through the ‘cavity’ module, the ‘build’ module considers a number of other inclusive parameters such as lead optimization, fragment linking, design mimicking, binding-affinity estimation, ligand filtering, ligand recommendation, synthesis analysis, substructure search, molecule clustering, etc. To generate the molecules, the LigBuilder uses three modes, namely, ‘growing,’ ‘linking,’ and ‘exploring.’ In the current study, the exploring mode was used to automate the generation of hRRM1 inhibitors. In short, the 3D coordinates of hRRM1 and the best docked gemcitabine structures were given as inputs. The ‘cavity’ module explored potential pharmacophoric features by using the key interactions site at the active site of hRRM1. Following the available pharmacophoric features, the ‘build’ module generated the set of molecules with a given number of parameters, including the number of generations, the population size, and the maximal number of outputs. After the successful generation of the molecular dataset, it was carefully checked for structural duplicity and similarity or dissimilarity before further analysis.

### 2.3. Molecular Docking Using Autodock Vina

Molecular docking is an effective and widely used molecular screening tool based on binding energy and interactions. Molecules generated from the de novo design approach were used for molecular docking to select compounds having a better binding affinity towards hRRM1. To use any docking methodology to dock unknown molecules, it is necessary to validate the docking protocol before executing it. The main objective of the docking protocol validation is to find the docking parameters that can reproduce a similar orientation as the experimental conformation of the molecule. In the current study, the self-docking approach was used to validate the docking protocol. In this approach, the co-crystal GDP was re-drawn and docked at the same position where it was bound. The best docked pose was superimposed on the co-crystal conformer of the GDP and the RMSD was recorded. The above procedure was repeated by changing the docking parameters such as the size and coordinates of the grid to optimize the docking parameters. It has been reported that the docking protocol gives RMSD ≤ 2 Å between the docked and co-crystal ligand and may be capable enough of reproducing a conformation similar to the crystalized orientation [76]. Considering the validated docking parameters, the molecular docking of de novo designed molecules along with gemcitabine and NSAH was performed. Before docking, the entire set of designed molecules along with gemcitabine and NSAH was prepared using OpenBabel [77] and Python RDKit [78]. Both are publicly available molecular file format conversion and preparation tools. Followed by the removal of redundant compounds all molecules were converted into a 3D format. The Gasteiger charge [79] was added and protonated at a pH of 7.4. Finally, all molecules were converted into .pdbqt format for the docking in ADV. From the docking validation procedure, the grid coordinates at the active site were considered to be (−22.188, 16.448, 24.853) along the x-, y-, and z-axes, respectively, with a grid size of 60 × 60 × 60. After successful molecular docking of the entire dataset, the binding energy of each molecule was explored. To select better hRRM1 affinity molecules, the highest negative binding energy among gemcitabine and NSAH was considered to be the threshold value. Molecules found to have better affinity towards hRRM1 in comparison to the threshold were taken into consideration for the next step of the assessment.

### 2.4. Unsupervised Pharmacophoric Features Assessment and Negative Image-Based Modeling

#### 2.4.1. K-Means Clustering

Unsupervised learning algorithms are used only when there is an input without any reference to their labels (i.e., unlabeled data) or if the relationship between the observations or outcome is unknown. Additionally, unsupervised algorithms improve the clustering process by using distance metrics and centroid points as constraints. This is helpful in identifying clusters that are linked to a particular target. *K*-means clustering is one of the most popular and straightforward unsupervised methods available. It needs a definite number of clusters (*k)*, which is the number of centroids needed in the dataset. A centroid is a location that represents the center of the cluster. Each cluster is allocated a data point using Euclidean distance by minimizing the in-cluster sum of squares. In other terms, the *k*-means (average) algorithm identifies the *k* number of centroids and then allocates every data point to the nearest cluster while keeping the centroids as small as possible. The *k*-means clustering algorithm [80] is given in Algorithm 1. In the current study, eight different pharmacophore properties such as hydrogen bond (HB) donor (HBD), HB acceptor (HBA), hydrophobe (HY), ring aromatic (RA), ionizable, lumped hydrophobe, negative ionizable and Zn binder for the six known hRRM1 inhibitors, and the entire set of molecules remained after the molecular docking-based screening was calculated using the Python RDkit. The calculated pharmacophore features can be used to form clustering groups. In order to predict the active compounds using unsupervised learning, the *k*-means clustering method was employed (active was considered as ‘1′ and inactive as ‘0′, and hence *k* = 2). The algorithm selects the centroids randomly, which are used as the initial points for each cluster, and then performs iterative calculations to optimize the centroids’ positions. The cluster creation or optimization is stopped when either (i) the centroids have stabilized because the clustering has been successful—there is no change in their values (convergence = 1 × 10^−4^); or (ii) the defined maximum number of iterations (1000) has been achieved. The following is the pseudo-code for the *k*-means clustering algorithm:
**Algorithm1.** The *k*-means clustering algorithm$Initialize\;n_{i},i=1,\ldots k,to\;k\;random\;x^{t\;}$ *Repeat* $For\;all\;x^{t}\;\in \;X$ $\;b_{i\;\leftarrow \{\begin{array}{c} 1\;if\left|\left|x^{1}-n_{i}\right|\right|=min_{j}\left|\left|x^{t}-n_{j}\right|\right|\\ 0\;otherwise \end{array}}^{t\;}$ $\mathrm{For}\;\mathrm{all}\;n_{i},i=1,\ldots ,k$ $\;n_{i}\leftarrow \sum_{t}b_{i}^{t}x^{t}/\sum_{t}b_{i}^{t}$ $\;Until\;m_{i}\;converge$

#### 2.4.2. Negative Image-Based Modeling

NIB screening is one of the geometry-optimized molecular docking approaches that considers both key information of receptor cavity and bound ligand, and, subsequently, the negative image is built based on shape and electrostatic parameters. PANTHER [81], a cavity detection tool, was used to develop the NIB models from the gemcitabine-bound receptor cavity of hRRM1. The radius size of the active site was considered to be 10 Å. A total of three NIB models was developed by using the docked gemcitabine at the centroid, defining the ligand distance limit of 1.2 Å, and body-centered cubic (bcc) packing. The center of the binding site was considered as it was considered in molecular docking at (−22.188, 16.448, 24.853) along the x-, y-, and z-axes, respectively. By considering the above center of the binding site, the negative image was created by pinning the shape/electrostatics or charge features of the ligand-binding cavity. To check the predictive ability of each model, a set of six known drugs/standard molecules for hRRM1 was used to calculate the ShaEP score by mapping through the ShaEP similarity tool. The total similarity score in the range of 0 to 1 was calculated by the ShaEP tool through the shape and electrostatic potential (ESP) of the NIB model. During the execution of scoring, equal weights were given for both shape and ESP. The correlation between inhibitory activity and ShaEP score was calculated for all three models. The best predictive model was further used to predict the ShaEP score of molecules that remained after molecular docking. The user-defined arbitrary ShaEP score was used to wipe out the low-affinity molecules.

Molecules designated as active in *k*-means clustering and ShaEP score better than the threshold were carefully checked, and common compounds found in both of the above approaches were considered for further assessment. The NIB package was developed by the University of Jyvaskyla, Jyvaskylan yliopisto, Finland.

### 2.5. Pharmacokinetics, Drug-Likeness, and Toxicity Assessment

De novo designed common molecules retained from both *k*-means clustering and the NIB model approach were used for the calculation of pharmacokinetic and drug-likeness properties using SwissADME [82], an online freely available tool. To check the drug likeness and ADME profile, several parameters including gastrointestinal (GI) [83] and blood–brain barrier (BBB) [83] absorption, solubility [83], Lipinski’s rule of five (LoF) [84], and Veber’s rule [85] were explored. In particular, the molecules found to be absorbed by the GI and not by the BBB along with highly soluble molecules were considered for further analysis. LoF explains that a molecule might possess drug likeness if the molecular weight (MW), hydrophobicity (logP), HBA, and HBD are not more than 500, 5, 10, and 5, respectively. Veber’s rule explains that being a drug, a molecule should have a number of rotatable bonds (NRB) ≤ 10 and a polar surface area ≤ 140 Å^2^.

Molecules following the ADME and drug-likeness characteristics were further used for toxicity assessment. *pkCSM* [86], an online tool, was used to extract several toxicity parameters including AMES toxicity, maximum recommended tolerated dose (MRDT), minnow toxicity (MT), and skin sensitization (SS). AMES explains mutagenic characteristics of the molecule and gives a positive or negative indication, whereas MT is known as LC_50_ [87] and represents the concentration of the molecule required to cause the death of 50% of flathead minnows. A molecule having an LC_50_ value of less than 0.5 mM is considered to be highly acutely toxic in nature. MRTD is the threshold toxic dose of the molecule in humans, and an MRTD ≤ 0.477 log(mg/kg/day) is considered to be a low dose. SS determines any adverse effect on the skin after taking the molecule and indicates either positive or negative. 

Molecules that satisfied pharmacokinetic, drug likeness, and toxicity parameters were carried forwarded for quality assessments. 

### 2.6. Molecular Docking Using PLANTS and SwissDock, and Absolute Binding Free Energy Calculation Using K_DEEP_

PLANTS [65], molecular docking software, uses the ant colony optimization (ACO) [88] method to dock the small molecules in the protein receptor site, and it was developed by the University of Konstantz, Germany. The fundamental concept of the method is that ants leave pheromones on the ground after finding food substances. Following the pheromones, other ants of the same species start chasing the trails with a higher concentration of pheromones and subsequently increase the pheromone layer on the trail [89]. The PLANTS tool follows the MIN–MAX ACO algorithm [90] in which each virtual ant identifies a solution to a general function defining the problem. The empirical scoring function further evaluates the solution, with a high value of the pheromone parameter receiving the best solution [91]. For the next iteration, during the ants' search for the solution, the values of the pheromone parameters for all the variables are considered [90]. SwissDock is an open source web server-based molecular docking engine, and it can be accessed at http://www.swissdock.ch/ (accessed on 3 September 2022). This webserver is developed and maintained by the Swiss Institute of Bioinformatics, Lausanne, Switzerland. Molecules obtained after toxicity analysis were considered for molecular docking using the PLANTS tool and SwissDock web server, and the binding energy from both the docking engines of each molecule was recorded.

Further, the above set of molecules was used for calculation of absolute binding free energy through K_DEEP_ [66], an online publicly available tool, which can be accessed through https://www.playmolecule.com/Kdeep/, Barcelona, Spain (accessed on 20 June 2022). It is based on the machine learning (ML) approach, such as 3D-convolutional neural networks (3D CNNs), for predicting protein−ligand absolute binding affinity. It is important to note that K_DEEP_ is pre-trained, tested, and validated through the PDBbind v.2016 database, Shanghai, China (accessed on 28 June 2022). This tool takes the input of the receptor file and the set of small molecules of which the absolute binding free energy needs to be calculated. In the current study, prepared hRRM1 target protein and de novo molecules obtained after toxicity assessment were given as inputs, and the remaining parameters were kept as default. Based on the pharmacophoric features (HBA, HBD, RA, HY, metallic, positive or negative ionizable, and total excluded volume) of proteins and ligands, it gave the 3D voxel representation of the binding site. Using the 3D CNN algorithm and pharmacophoric features, the models were generated, and subsequently these models were used for the absolute binding free energy calculation.

### 2.7. Pharmacophoric Features Assessment

One of the decisive variables for being a good binder at the active site is the existence of essential pharmacophoric characteristics in any small molecule. Therefore, four prominent pharmacophoric features, namely, HBA, HBD, HY, and RA, were extracted using Python RDKit [78] from the de novo designed molecules reserved after toxicity analysis along with gemcitabine and NSAH. A comparative analysis of the pharmacophoric features was carried out between proposed hRRM1 inhibitors, and gemcitabine and NSAH. The pharmacophore fraction was computed to have a better understanding of the role of the pharmacophoric functions in the proposed compounds compared to standard BTC molecules.

In particular, the combined pharmacophore fraction (*cPharmFrac*) and pharmacophore fraction (*PharmFrac*) were estimated from merged gemcitabine and NSAH, and proposed hRRM1 inhibitors, respectively. The *cPharmFrac* of both standard molecules was calculated by division of the total number of specific pharmacophoric features present in both molecules by the total number of all pharmacophoric features of gemcitabine and NSAH. In the case of the de novo designed molecule, the *PharmFrac* was estimated by dividing the number of specific pharmacophoric features by the total number of features of the particular molecule. The following expressions were used to calculate the *cPharmFrac* and *PharmFrac*:

(1) $$cPharmFrac=\frac{\sum S_{pharm}}{n_{sf}}$$

(2) $$PharmFrac=\frac{P_{pharm}}{n_{pf}}$$

*S_pharm_* represents the pharmacophoric features (HBA, HBD, HY, and RA) of standard molecules such as gemcitabine and NSAH. The *n_sf_* defines the total number of all pharmacophoric features in both the standard molecules. *P_pharm_* signifies the total number of individual pharmacophoric features (i.e., HBA, HBD, HY, and RA are considered individually) of a particular proposed molecule, and *n_pf_* denotes the total number of features present (i.e., summation of HBA, HBD, HY, and RA) in the proposed molecules. 

### 2.8. Molecular Dynamics Simulation

All-atom MD simulation is an excellent and widely used approach to explore the behavior of the ligand–protein complex in dynamic states immerged in explicit water molecules. Initially, the stability between the molecules retained after toxicity along with gemcitabine and NSAH, and hRRM1 complexes were assessed through a short time span MD simulation of 10 ns. Based on the statistical parameters and MM-GBSA-based binding affinity, top-ranked molecules, gemcitabine and NSAH bound with hRRM1, were further extended up to 100 ns. The MD simulation was carried out in GROningen MAchine for Chemical Simulations (GROMACS) v2021.2 [92,93], an open source software tool developed by Groningen University, Groningen, Netherlands The experiment was performed with a time step, constant pressure, and constant temperature of 2 fs, 1 atm, and 300 K, respectively. The topology of hRRM1 was generated through an all-atoms CHARMM36 forcefield [94]. The proposed hRRM1 inhibitors, and gemcitabine and NSAH were considered in SwissParam [95] to obtain the topology and other parameter files. The protein–ligand complexes were immerged into the cubic box with a minimum distance of 10 Å from the center to the box edge. The system was solvated using the transferable intermolecular potential with a 3 points (TIP3P) [96] water model. A number of required Na^+^/Cl^−^ ions was added to neutralize each of the systems. The steepest-descent algorithm was used to minimize each system for addressing the close contacts or overlaps between the atoms. To equally distribute the water molecules and ions around the system, each of the systems was equilibrated through NVT (constant number of particles, volume, and temperature) followed by NPT (constant number of particles, pressure, and temperature). Upon successful completion, the MD simulation trajectories were used to calculate a number of parameters including hRRM1 backbone RMSD, ligand RMSD, root-mean-square fluctuation (RMSF), radius of gyration (RoG), and the number of inter-molecular HBs.

### 2.9. Binding Free Energy Using MM-GBSA Approach and Per-Residue Decomposition Energy Calculation

The binding free energy (*ΔG_bind_*) calculated using the MM-GBSA approach from MD simulation trajectories is considered to be more authentic and widely acceptable in comparison to the binding energy derived in the molecular docking study. Moreover, it is computationally efficient and considered to be a better estimation in comparison to the several scoring functions. From the MD simulation trajectories, the (*ΔG_bind_*) of each of the final hRRM1 inhibitors along with gemcitabine and NSAH were calculated using the gmx_MMPBSA module [97]. A total of 2000 frames from the entire trajectory was considered for (*ΔG_bind_*) estimation. The following expressions were used to calculate the *ΔG_bind_*:

(3) $$\Delta G_{\mathrm{bind}}=\langle G_{\mathrm{complex}}\rangle \;-\;\langle G_{\mathrm{receptor}}\rangle \;-\;\langle G_{\mathrm{ligand}}\rangle$$

where G_complex_, G_receptor_, and G_ligand_ are the binding energy of the protein–ligand complex, receptor, and ligand, respectively.

The *ΔG_bind_* can also be expressed as

(4) $$\Delta G_{\mathrm{bind}}=\Delta H\;-\;T\Delta S$$

ΔH is the enthalpy of binding, whereas TΔS represents the conformational entropy after ligand binding. On the removal of the entropic term, the estimated value represents the effective free energy [98]. The effective free energy is sufficient to compare the relative binding energy of any small molecule.

Further, the ΔH can be split into the following individual terms

(5) $$\Delta H={\Delta E}_{\mathrm{MM}}{+\Delta G}_{\mathrm{sol}}$$

where ΔE_MM_ can be expressed as a summation of bonded and non-bonded terms as below.

(6) $${\Delta E}_{\mathrm{MM}}{=\Delta E}_{\mathrm{bonded}}{+\Delta E}_{\mathrm{nonbonded}}$$

E_bonded_ represents the combination of three terms including bond stretching, angle bending, and torsion angle. The ΔE_nonbonded_ is the combination of electrostatic and van der Waals’ terms. Both expressions are given below.

(7) $${\Delta E}_{\mathrm{bonded}}{=\Delta E}_{\text{bond\_length}}{+\Delta E}_{\mathrm{angle}}{+\Delta E}_{\mathrm{dihedral}}$$

(8) $${\Delta E}_{\mathrm{nonbonded}}{=\Delta E}_{\mathrm{ele}}{+\Delta E}_{\mathrm{vdW}}$$

The solvation energy (ΔG_sol_) for GB models can be calculated using the polar constituent only. The nonpolar (NP) constituent is mostly thought to be proportional to the molecule’s total solvent accessible surface area (SASA), with a proportionality constant derived from experimental solvation energies of small nonpolar molecules [99,100]. Both solvation and non-polar energy terms are expressed as given below.

(9) $${\Delta G}_{\mathrm{sol}}{=\Delta G}_{\mathrm{polar}}{+\Delta G}_{\text{non-polar}}{=\Delta G}_{\mathrm{GB}}{+\Delta G}_{\text{non-polar}}$$

(10) $${\Delta G}_{\text{non-polar}}{=\mathrm{NP}}_{\mathrm{TENSIO}}{}_{N}{+\Delta }_{\mathrm{SASA}}{+\mathrm{NP}}_{\mathrm{OFFSET}}$$

Upon successful calculation of the *ΔG_bind_* of each molecule, it was documented along with the standard deviation.

Dynamic changes of the amino acids present around the active site of any protein target play a key role in holding the ligand. The contribution of amino acids toward the ligand for the binding interaction formation may be explored through per-residue decomposition energy. A total of 2000 frames from the whole trajectory was used to calculate the per-residue decomposition energy of hRMM1 amino acids around 5 Å from the final molecules [101].

## 3. Results and Discussion

A comprehensive computational molecular design and optimization protocol was implemented to identify potential novel hRRM1 inhibitors/modulators for the treatment and/or management of BTC. To find out novel and effective compounds against disease-specific macromolecular targets, the computational drug discovery pipeline has already gained gigantic momentum in more than the last three decades.

**Figure 1:**
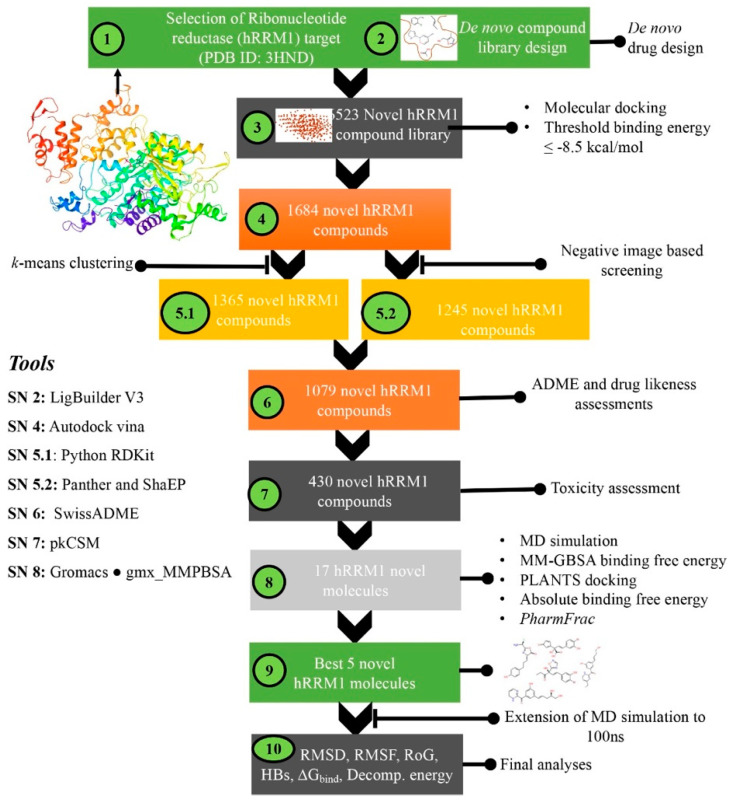
Schematic workflow for designing hRRM1 inhibitors using de novo drug design and virtual screening approaches.

Herein, a number of pharmacoinformatics approaches including de novo molecular design technique, followed by molecular docking, NIB screening, pharmacophoric features assessment, ADME, toxicity, machine learning (ML)-based absolute binding affinity estimation, MD simulation, and MM-GBSA-based binding free energy estimations were employed and executed for sequentially filtering out some potent drug-like candidates for hRRM1 target protein. The entire stepwise schematic workflow of the employed work is given in Figure 1. Validation of the molecular docking methodology is an essential step before it can be used to screen any molecular dataset, whether it be an existing database or a newly built molecule. Therefore, in the current study, the self-docking approach was initially adopted to check the reproducibility of the comparable orientation of the co-crystal GDP through a molecular docking study. The re-drawn GDP was docked at the same position of the active site of hRRM1 where the co-crystal GDP was bound. The best-docked pose was extracted and superimposed on the original co-crystal conformer of GDP. To explore the orientational similarity between the best-docked pose and the original crystal conformer, the RMSD was calculated and found to be 1.235 Å. In the molecular docking study, the observed RMSD (<2 Å) indicated that the conformational orientation of the docked pose was found to be almost similar to co-crystal-bound GDP. Hence, it can be postulated that if any new or unknown molecule is docked in hRRM1 through the above docking parameters, it will be able to produce possibly similar orientational conformations as the crystallized ligand of hRRM1. The superimposed docked pose and co-crystal conformer is given in Figure S1 (Supplementary Data).

### 3.1. De Novo Design of hRRM1 Inhibitors and Virtual Screening

Gemcitabine was initially docked in the active site of hRRM1 (PDB ID:3HND) through ADV, and the binding energy was found to be −8.50 kcal/mol. Out of nine docked poses, the best pose was selected through the binding energy and binding interaction analyses. The above complex of hRRM1 and gemcitabine was used to design the novel molecules through the de novo design approach. Upon successful completion of all 10 given sessions of jobs, a total of 10,000 new molecules was designed based on the features' active site cavity. For all those newly designed molecules, the molecular frameworks such as structural organization and bad valence error representation were checked. Few newly designed molecules may have structural similarities due to the usage of the same receptor cavity during molecule generation. As a result, duplicate or identical molecules were removed prior to further analysis. A total of 3477 de novo designed molecules was found to be identical and hence were eliminated from the dataset. The remaining 6523 unique hRRM1 molecules were taken into consideration for further assessment. On close inspection, it was observed that molecules were structurally diverse in nature, having a wide variety of scaffolds. Almost all of the molecules consist of several pharmacophoric features that might be crucial for the interactions with hRRM1 active site amino acid residues.

#### 3.1.1. Molecular Docking Based Screening

The entire set of molecules obtained through the de novo design approach along with gemcitabine and NSAH were considered for the molecular docking in ADV. From the molecular docking study, it was revealed that almost each and every molecule showed a significant binding affinity towards hRRM1, having a binding energy range of −5.10 to −13.80 kcal/mol. The binding energy values of gemcitabine and NSAH were found to be −7.20 and −8.50 kcal/mol, respectively. To reduce the chemical space of docked de novo designed inhibitors, the threshold binding energy was set to −8.50 kcal/mol. The main motive behind such consideration was to select molecules that show better binding affinity towards hRRM1 in comparison to gemcitabine and NSAH. Applying the above criteria, it was found that 4839 compounds failed to show better binding affinity towards the hRRM1 and hence were removed from further evaluation. The remaining 1684 novel inhibitors/modulators were further considered for a number of pharmacophoric feature assessments based on the *k*-means clustering approach and followed by other filtration techniques including NIB screening, pharmacokinetics and toxicity analyses, quality checking through PLANTS docking, ML-based absolute binding free energy, MD simulation, and MM-GBSA-based binding free energy.

#### 3.1.2. K-Means Clustering of Pharmacophoric Features

The presence of suitable pharmacophoric features in any small molecule is very crucial for showing strong binding affinity with active site amino acid residues of the target protein. The pharmacophoric pattern in de novo designed molecules obtained after the molecular docking study was explored through an unsupervised *k*-means clustering algorithm. In this method, a set of known active BTC drug/standard molecules was considered as a training set, and eight pharmacophoric features of each molecule were calculated through Python RDKit. Further, the same set of pharmacophoric features for 1684 de novo designed inhibitors was extracted. Based on pharmacophoric features of drug/standard molecules and exploring the features of test compounds, the de novo designed molecules that remained after the docking study were classified into active and inactive classes. A set of 1365 molecules was found to be active and considered for further assessment.

#### 3.1.3. Negative Image-Based Screening

**Figure 2:**
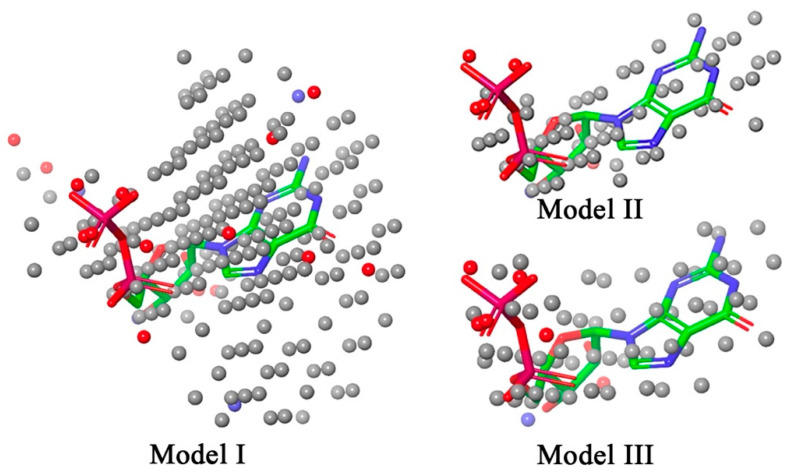
The negative image-based models developed for the receptor of hRRM1. The GDP is mapped in each generated NIB model.

The GDP bound complex of hRRM1 was used to develop the NIB models by considering the geometry and electrostatic characteristics of the receptor cavity. Three models (**Models I**, **II,** and **III**) were generated by considering the GDP as the centroid, limiting the model generation to 1.5 Å around the GDP and bcc cubic packing, respectively. All three generated models were found to be perfectly mapped with the co-crystal GDP and are given in Figure 2.

It was essential to evaluate the predictivity and validity of the NIB model before using the prediction on the unknown molecular dataset. The ShaEP score of a set of known active hRRM1 inhibitors was estimated and is given in Table 1. Upon close observation, it can be seen that the ShaEP score was found to be in the range of 0.513 to 0.720. Precisely, the lowest and highest ShaEP scores of the hRRM1 inhibitors' scores were observed to be 0.307 and 0.654, 0.513 and 0.681, and 0.454 and 0.645 for **Models I**, **II,** and **III,** respectively. It is interesting to see that not a single molecule predicted a ShaEP value less than 0.5 after estimation using **Model II**. Further, to check the quality of the prediction of each model, the correlation coefficient (*R*) between the ShaEP score and logarithm value of the experimental *IC_50_* was calculated for all three models and is given in Table 1. It can be seen that **Model II** had the highest *R* value of 0.660, followed by **Model III** and **Model I** with 0.535 and 0.480, respectively. The above data and observations undoubtedly suggested that the prognostic power of **Model II** was better than the other two models. Hence, **Model II** was selected for utilization of the ShaEP score-based screening of molecules that remained after the molecular docking study. 

Molecules that remained after the molecular docking binding energy-based screening (a total of 1684 compounds) were mapped on **Model II**, and the ShaEP score was calculated. Upon detailed analysis, it was revealed that the ShaEP score was found to be in the range of 0.231 to 0.857. Moreover, most of the molecules almost perfectly occupied the entire part of the model. To select better-fitted molecules, arbitrarily the ShaEP score of 0.6 and more was considered as a threshold. It is important to note that the ShaEP scores of both standard molecules were found to be less than 0.530. Hence, molecules that were shown to have a ShaEP score ≥ 0.6 were taken for further evaluation. A set of 1245 molecules satisfied the above criteria. 

The common molecules obtained from both methodologies, such as *k*-means clustering of pharmacophoric and NIB screening, were used to investigate the next level of assessments, and it was found that 1079 molecules were retained. 

#### 3.1.4. Pharmacokinetics and Drug-Likeness Parameter Assessment

In the computational drug design paradigm, pharmacokinetics and drug likeness parameters evaluation have become essential aspects to screen potential small molecules with safer ADME and better medicinal chemistry properties. In the current study, molecules that retained after *k*-means clustering of pharmacophoric features and NIB screening were considered for the calculation of various ADME parameters. The SwissADME tool was used for the calculation of the pharmacokinetic properties of a total of 1079 molecules. Several parameters including GI absorption = yes, BBB = No, solubility = high, TPSA ≤ 140 Å^2^, and violation of LoF and Veber’s rule, i.e., as 0 (zero), were implemented to reduce the chemical space. After implementing the above rules, it was found that 649 molecules failed to follow at least one of the rules and hence were removed for further assessment. Hence, the remaining 430 molecules were used for the next level of screening to wipe out the inactive molecules.

#### 3.1.5. Toxicity Assessment

**Figure 3:**
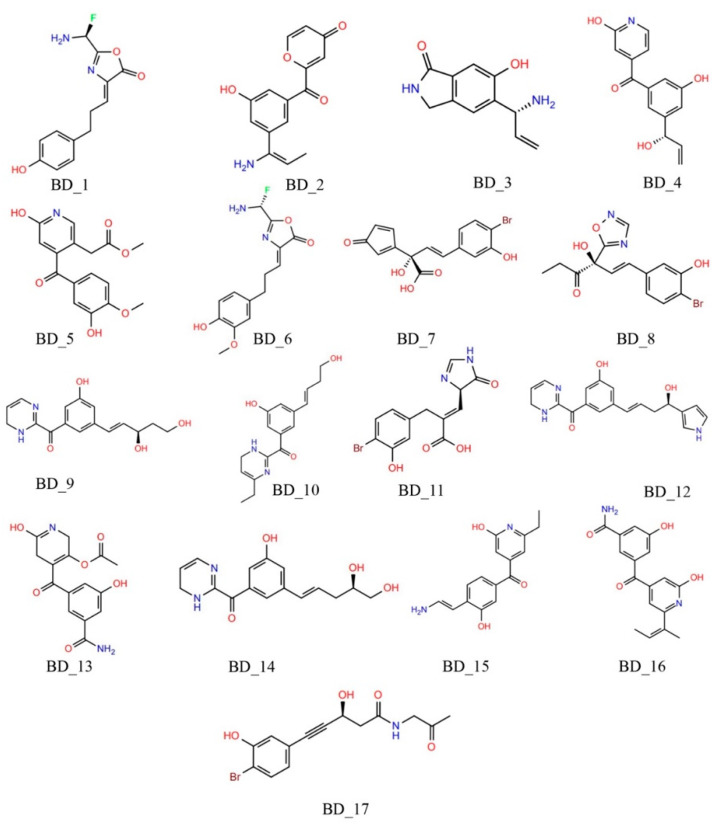
Two-dimensional representation of the final 17 de novo designed molecules for BTC.

Molecules retained after the ADME analysis were further used for the calculation of the toxicity parameters using *pkCSM*, a widely used toxicity prediction online tool. Molecules found to have hepatotoxic and mutagenic (AMES toxicity) characteristics were removed. The extreme toxic dose, i.e., MTD, and lethal concentration (minnow toxicity) values of each molecule were checked, and compounds beyond the acceptable range (>0.477 and <0.5 mM, respectively) were deleted. Finally, skin allergic compounds were identified using the skin sensitization parameter. Molecules having non-allergic characteristics were considered for further analysis. A total of 17 molecules persisted after screening through the above parameters and was taken into consideration for the MD simulation study. The two-dimensional (2D) representation of the 17 molecules is given in Figure 3. 

#### 3.1.6. Molecular Docking Using PLANTS and SwissDock, and Absolute Binding Affinity Calculation through K_DEEP_

A total of 17 molecules was found to be safe and free from any toxic nature and therefore was further considered for the quality assessment through molecular docking in PLANTS. Following docking, the absolute binding affinity calculation was also carried out using K_DEEP_. PLANTS is the ACO-based docking engine that samples the search space. The same parameters of grid configuration, i.e., active site coordinates and grid size, used in the ADV docking study were considered in PLANTS docking. After successful docking, the PLANTS score was recorded, and it is given in Table 2. The PLANTS scores of the hRRM1 inhibitors were found to be within the range of −61.00 to −93.00 kcal/mol. On the other hand, the PLANTS scores of both standard molecules, gemcitabine and NSAH, were found to be −67.106 and −80.290 kcal/mol, respectively. It is interesting to note that except for BD_13, all other molecules were found to have better binding affinity in comparison to gemcitabine. When the PLANTS scores were compared to the NSAH, it was discovered that BD 1, BD 6, BD 7, BD 8, BD 9, BD 12, BD 14, and BD 17 had a stronger binding affinity for the hRRM1. The rest of the compounds were discovered to have a binding affinity similar to NSAH. For further cross check, all the above molecules were docked using the SwissDock server, and ΔG (kcal/mol) was recorded and is given in Table 2. The ΔG values of gemcitabine and NSAH were found to be 7.46 and 7.19 kcal/mol, respectively. It is important to note that all the proposed hRRM1 molecules were found to have better binding affinity in comparison to NSAH. On the other hand, except for BD_4 and BD_5, all other molecules showed higher binding affinity in comparison to gemcitabine. The above observations from the SwissDock study clearly corroborated the outcomes from PLANTS and ADV.

The best-docked posed of all 17 molecules along with gemcitabine and NSAH obtained from the PLANTS docking study were considered for the calculation of absolute binding affinity towards hRRM1 through K_DEEP_. The absolute binding affinities of all the molecules are given in Table 2. Upon close observation of the absolute binding affinities, it was revealed that all 17 de novo designed molecules were found to have better binding affinities in comparison to both gemcitabine and NSAH. In detail, the absolute binding affinities of gemcitabine and NSAH were recorded as −5.819 and −5.520 kcal/mol, respectively. Among the de novo designed molecules, the highest and lowest absolute binding affinities were found for BD_16 and BD_3, respectively. K_DEEP_-based absolute binding affinity data suggested that most of the identified molecules possess a strong binding affinity towards hRRM1. 

Overall, a few molecules were also found to show a slightly lower binding affinity in comparison to standard molecules toward hRRM1. At the same time, all molecules were found to have strong interaction affinities through absolute binding affinity analysis. The molecules found with slightly less affinity were closely checked and found to show high negative absolute binding affinities in K_DEEP_. The chemical space could be reduced based on the PLANTS score, but it would violate the K_DEEP_ binding affinity assessment. Hence, without removing any molecules for further evolution, all 17 molecules were carried forward for pharmacophoric features exploration followed by MD simulation and binding free energy estimation using the MM-GBSA approach.

#### 3.1.7. Pharmacophoric Features Assessment

The optimal position and arrangement of pharmacophoric features can give crucial binding interactions that might lead to achieving the active conformation of the molecule with a better affinity towards the protein target. All 17 proposed hRRM1 inhibitors along with gemcitabine and NSAH were considered for the estimation of available pharmacophoric features such as HBD, HBA, HY, and RA through Python RDKit [78], and the results are given in Table S1 (Supplementary File). Gemcitabine and NSAH were found to have values of 3 and 3, 5 and 3, 0 and 4, and 1 and 3 of HBD, HBA, HY, and RA, respectively. The total numbers of pharmacophoric features present in gemcitabine and NSAH were found to be 9 and 13, respectively. Interestingly, all 17 de novo designed molecules contained a higher number of pharmacophoric features in comparison to gemcitabine. On the other hand, except for BD_1, BD_2, BD_3, and BD_6, all other hRRM1 inhibitors were found to hold a higher number of pharmacophoric features than NSAH. Further, to better understand the presence of pharmacophoric features in the molecules, the *cPharmFrac* and *PharmFrac* were calculated using Equations (1) and (2), respectively, and the results are given in Table 3. 

From Table 3, it can be observed that *PharmFrac* like HBD, HBA, and HY of almost all 17 molecules were found to be similar to or better than the *cPharmFrac*. It is important to note that all de novo designed molecules were seen to have a higher contribution of HY *PharmFrac* in comparison to *cPharmFrac*. The above observation suggested that molecules possessed sufficient chemical functionality to form potential HB and hydrophobic interactions with active site amino acid residues of hRRM1. 

#### 3.1.8. MD Simulation and MM-GBSA Binding Free Energy Estimation

To explore the more rigorous conformational stability and binding affinity of each of the 17 molecules towards hRRM1, a short MD simulation of 10 ns was carried out. To compare the MD simulation data and binding free energy of the de novo designed molecules, both standard molecules, gemcitabine and NSAH, were also taken for MD simulation. A number of stability determining parameters such as protein backbone and ligand RMSD, RoG, and intermolecular hydrogen bonds were analyzed. All the above data are given in Figures S2–S5 (Supplementary File). Each of the protein backbone RMSD, ligand RMSD, and RoG parameters was found to explain the stability of the protein–ligand complexes with some variations. The inter-molecular HBs in almost each of the 17 molecules were found to be comparable with gemcitabine and better than NSAH. Further, the binding affinity of each molecule along with gemcitabine and NASH was assessed through the calculation of binding free energy (*ΔG_bind_*) using the MM-GBSA approach, and it is given in Table 4. High negative *ΔG_bind_* reflects the higher binding affinity of the molecules towards hRRM1. It can be seen that the *ΔG_bind_* values of gemcitabine and NASH were found to be –36.65 and −19.53 kcal/mol, respectively. As seen in Table 4, it was observed that BD_7 and BD_8 showed better binding affinity in comparison to both gemcitabine and NSAH. Further, BD_1, BD_10, and BD_14 were found to have *ΔG_bind_* values of −24.62, −27.02, and −29.75 kcal/mol, respectively. The binding affinities of the above three molecules were found to be better than NSAH and comparable to gemcitabine. Although BD_6 showed a slightly better binding energy in comparison to NSAH, at the same time it was far from gemcitabine. The remaining molecules were found to have low binding affinities in comparison to both standard molecules.

From all the above data and analyses, it is nearly clear that the selected 17 molecules possess a strong competence of being potential hRRM1 inhibitors. The molecular docking through both ADV and PLANTS undoubtedly substantiated the high affinity of these molecules towards hRRM1. Unsupervised *k*-means clustering already approved these molecules as active in nature, and this was corroborated by high negative convolutional neural networks based on absolute binding free energy estimated through K_DEEP_. Moreover, the negative image of the active site cavity showed complete affection towards the molecules confirmed through a high ShaEP score. The contribution of significant *PharmFrac* for each molecule exposed their prospect of being a good binder at the hRRM1 receptor site. A number of statistical parameters calculated from a short MD simulation of each molecule bound with hRRM1 unfolded their compactness and convincing association in the dynamic states. High negative binding free energy calculated using the MM-GBSA method disclosed the strong affinity and their being promising compounds to modulate the hRRM1 activity. In general, it can be considered that all the 17 molecules in Figure 3 might be crucial for the modulation of hRRM1 activity and effective chemical entities for the management of BTC. Although it is very clear that all the molecules (in Figure 3) showed their potentiality, BD_1, BD_7, BD_8, BD_10, and BD_14 were found to have more affinity towards hRRM1. For further confirmation and exploration of the relative stability, the above five molecules along with gemcitabine and NSAH bound with hRRM1 were subjected to extended MD simulation up to 100 ns of time span. 

### 3.2. Binding Interaction Analysis

**Figure 4:**
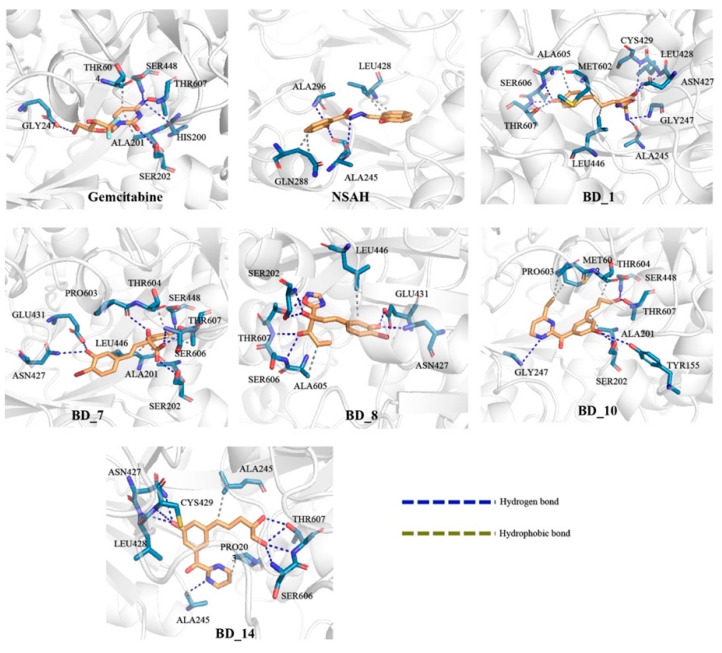
Binding interaction profile of top five molecules, gemcitabine, and NSAH.

The binding interaction analyses of the top five molecules, gemcitabine, and NSAH were explored, and it is given in Figure 4. Each of HIS200, SER202, GLU431, SER448, and THR607 were found to form HB interactions with gemcitabine separately. Additionally, ALA201 and THR604 established hydrophobic interaction with gemcitabine. Upon binding interaction analysis of NSAH with hRRM1, it was revealed that ALA245, GLY246, ARG293, and ALA296 were critically connected through HB interactions. NSAH also formed two hydrophobic connections with each of GLN288 and LEU428. BD_1 possesses a number of important chemical functional groups to form critical bonds with hRRM1 active site amino acid residues. The amine group present in BD_1 was seen to interact with ALA245 and GLY247 through HB interactions. ASN427, LEU428, and CYS429 of hRRM1 formed HB interactions with the oxo group attached to oxazolone of BD_1. The hydroxyl group attached to the phenyl ring of BD_1 was successfully connected with SER606 and THR607 through one and two HB interactions, respectively. Beyond the above, the phenyl ring and the unsaturated chain between phenyl and oxazolone rings were found to be important to impart hydrophobicity, which was confirmed by the formation of hydrophobic interactions with LEU446, MET602, and ALA605. The hydroxyl group attached to the phenyl ring in BD_7 critically established HB interactions with ASN427 and GLU431. The oxo group of cyclopentadienone was crucial to form HB interactions with SER448 and THR607. Both hydroxyl groups attached to the non-cyclic part of BD_7 interacted with SER202, SER606, and THR607 via HB interactions. Both cyclic rings such as phenyl and cyclopentadienone were found to be important for hydrophobic interactions with ALA201, LEU446, THR604, and THR607. A number of crucial binding interactions in terms of HB and hydrophobic contacts were found between hRRM1 amino acids and BD_8. Both the hydroxyl groups, the oxo group, and the nitrogen atom at the oxadiazole ring present in BD_8 participated in HB formation with SER202, ASN427, GLU431, SER606, and THR607. Moreover, both the unsaturated alkyl group and phenyl ring were critically found to be hydrophobic in nature and formed hydrophobic contacts with LEU446 and ALA605. Two hydroxyl groups of BD_10 were potentially formed HB with each of TYR155, SER202, SER448, and THR607, separately. Moreover, the NH– group of dihydropyrazine in BD_10 was also seen to connect with GLY247 through HB interactions. Moreover, saturated and unsaturated alkyl groups of BD_10 were found effective to form hydrophobic contacts with ALA201, MET602, PRO603, and THR604. Similar to the above molecules, BD_14 was also found to have a number of crucial chemical functional groups important for binding interactions with hRRM1 amino acid residues. A hydroxyl group attached with the phenyl ring was seen to form three HB interactions with ASN427, LEU428, and CYS429. Another two hydroxyl groups present at the alkyl terminal of BD_14 were critically formed in one and two HB interactions with SER606 and THR607, respectively. Both PRO203 and LEU446 of hRRM1 successfully established hydrophobic binding interactions with BD_14.

From the binding interaction analyses, it was found that SER202 and GLU431 were found to establish HB interactions in gemcitabine, BD_7, and BD_8. Similarly, SER448 was also found to interact with gemcitabine, BD_7, and BD_10. It is important to note that THR607 was found to interact with gemcitabine and all five proposed hRRM1 inhibitors. Both ALA201 and THR604 were seen to form hydrophobic contacts with gemcitabine, BD_7, and BD_10. The amino acids residue ALA245 was found to interact with both NSAH and BD_14. Hence, it was undoubtedly clear that all the five proposed molecules were found to show almost a similar binding interaction pattern in molecular docking simulation.

**Figure 5:**
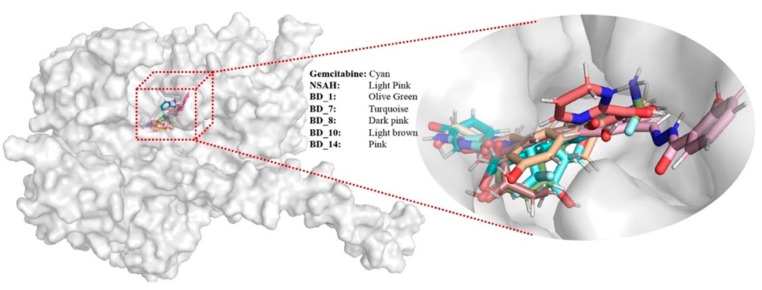
Binding mode in the 3D surface view of gemcitabine, NSAH, BD_1, BD_7, BD_8, BD_10, and BD_14 in hRRM1.

From the analysis of the binding interaction, it was very clear that all proposed molecules perfectly occupied the biologically relevant active site of hRRM1. For better observation, the surface view of all hRRM1 molecules including gemcitabine and NSAH was explored, and it is given in Figure 5. It is very clear that all molecules including gemcitabine and NSAH were bound in almost the same position of the hRRM1 active site cavity. Moreover, a number of common amino acids was also seen to interact with standard and proposed molecules. Hence, detailed binding interactions analysis and a buried view in 3D space successfully substantiated the potentiality and effectivity of the proposed molecules for hRRM1 activity modulation.

### 3.3. Molecular Dynamics Simulation of Top Five hRRM1 Inhibitors

For extensive exploration of the dynamic nature of the five best molecules along with gemcitabine and NSAH, the MD simulation was further extended up to 100 ns of time span. From the completed MD simulation trajectories, a number of statistical parameters including protein backbone RMSD, ligand RMSD, RMSF, RoG, and number of inter-molecular hydrogen bonds was extracted. The average, maximum, and minimum protein backbone RMSD, ligand RMSD, RMSF, and RoG are given in Table 5.

#### 3.3.1. Root-Mean Square Deviation

**Figure 6:**
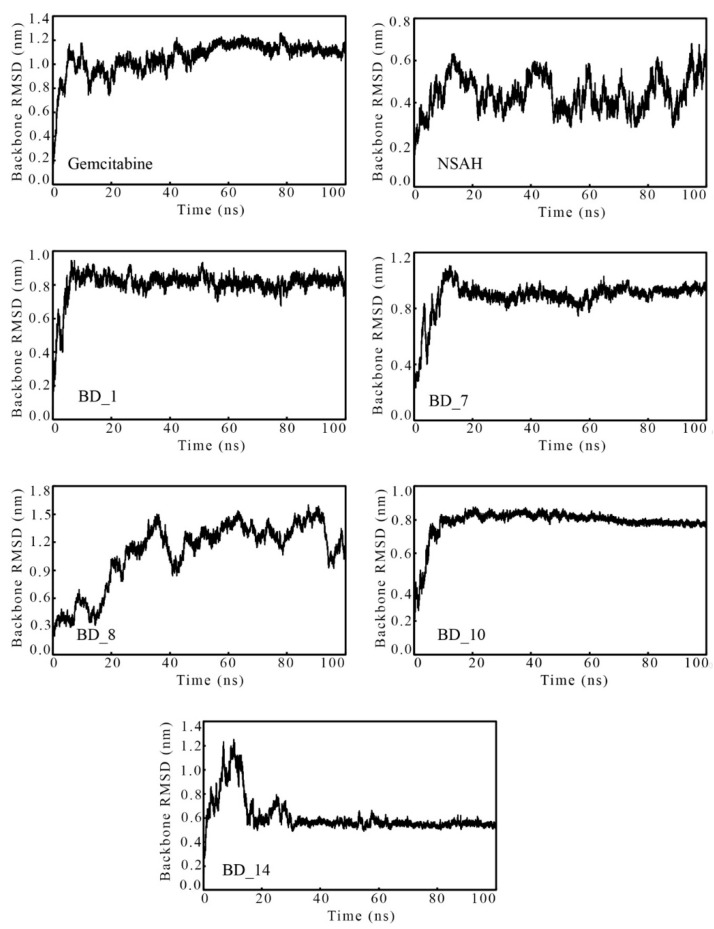
The hRRM1 backbone RMSD bound with gemcitabine, NSAH, and proposed hRRM1 inhibitors over time of simulation.

The protein backbone RMSD obtained from MD simulation trajectories is one of the important parameters to explore the insight protein backbone and ligand stability during the MD simulation. The consistent deviation or low variation of the RMSD value explains the consistency of the protein–ligand complexes in dynamic states. The RMSD of each of the 100,000 frames over the time of simulation was plotted and is given in Figure 6.

It was seen that the hRRM1 backbone bound with gemcitabine deviated slightly in the initial stage of simulation, and afterward it achieved consistency around an RMSD of 1 nm till the end of the simulation. Although the RMSD of the hRRM1 backbone bound with NSAH was found to be low, in the range of 0.44 to 0.69 nm, it was observed to deviate throughout the entire simulation. Among the top five proposed hRRM1 inhibitors, the hRRM1 backbone bound with BD_1, BD_7, and BD_10 was found to deviate in an almost similar manner at around 0.8 to 0.9 nm and remained consistent throughout the simulation. The hRRM1 backbone RMSD was found to deviate up to about 25 ns when bound with BD_14, and afterward it attained consistency at around 0.6 nm till the end of the simulation. It was unlikely that the hRRM1 backbone RMSD bound with BD_8 was found to deviate continuously throughout the simulation. It might be due to more conformational changes of BD_8 inside the hRRM1 binding pocket, resulting in consistent deviation of the backbone. The average hRRM1 backbone RMSD was found to be 1.057, 0.442, 0.806, 0.884, 1.092, 0.785, and 0.611 nm for gemcitabine, NSAH, BD_1, BD_7, BD_8, BD_10, and BD_14, respectively. The above value undoubtedly explained that the hRRM1 backbone RMSD of all proposed molecules was lesser than gemcitabine, except for BD_8. More deviation of the hRRM1 backbone bound with BD_8 might be the reason for having a slightly higher average RMSD. The lowest average hRRM1 backbone RMSD was found for NSAH, but the backbone was found to be a bit unstable in the MD simulation trajectory. Hence, from the above data and observations, it was clear that except for BD_8, the remaining proposed molecules bound with hRR achieved stability in the dynamic states.

**Figure 7:**
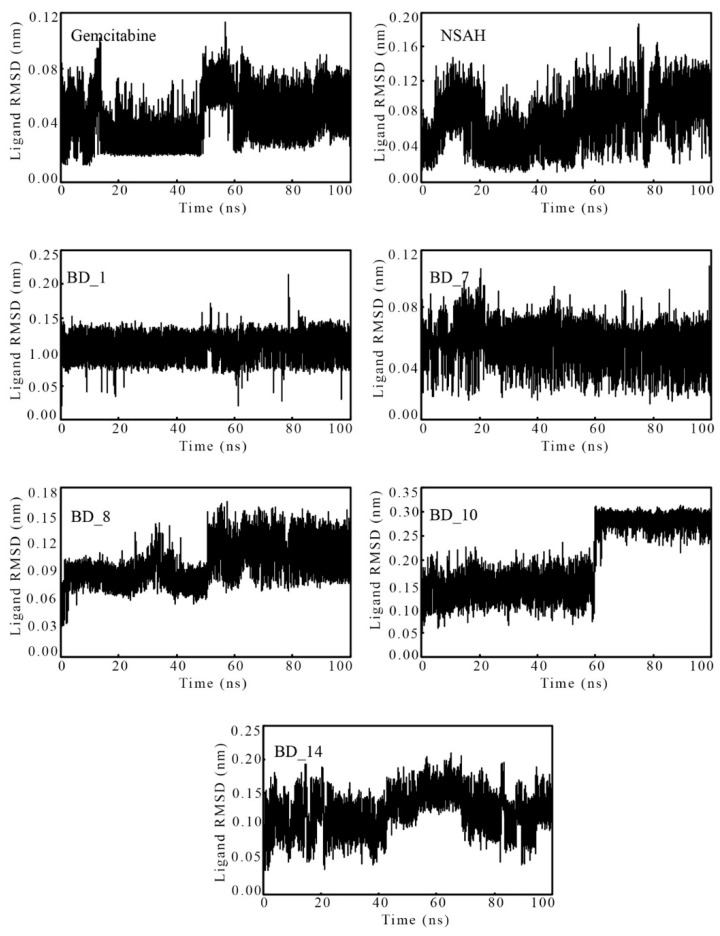
RMSD against the time of simulation of gemcitabine, NSAH, BD_1, BD_7, BD_8, BD_10, and BD_14.

The ligand RMSD data against the time of simulation of gemcitabine, NSAH, BD_1, BD_7, BD_8, BD_10, and BD_14 was plotted and is given in Figure 7. With some exceptions, almost all ligands remained steady in dynamic states throughout the simulation. BD_10 was seen to be coherent from the beginning to about 60 ns. Afterward, suddenly the RMSD was increased from around 0.15 to 0.30 nm and further attained steadiness till the end of the simulation. The above change might be due to a change in the conformational orientation of the molecule. The differences between the maximum and average RMSD can give an idea about the overall deviation of molecules from their mean position, and they were found to be 0.070, 0.115, 0.107, 0.056, 0.066, 0.114, and 0.086 nm for gemcitabine, NSAH, BD_1, BD_7, BD_8, BD_10, and BD_14, respectively. The above low values along with a consistent variation of ligand RMSD suggested the steadiness of the molecules inside the active site cavity of hRRM1.

#### 3.3.2. Root-Mean Square Fluctuation

**Figure 8:**
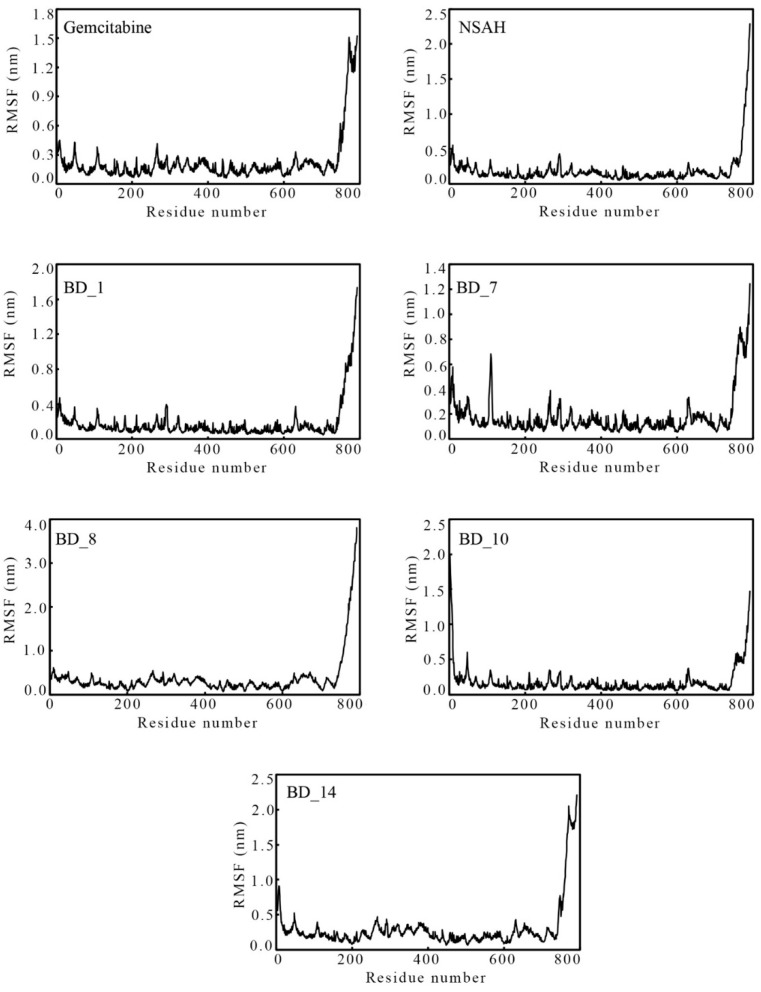
RMSF of individual hRRM1 amino acids bound with gemcitabine, NSAH, BD_1, BD_7, BD_8, BD_10, and BD_14.

The individual amino acids play a critical role in the stability of the protein and ligand complex in the MD simulation. Based on the deviation or conformational changes of bound small molecules, the amino acid residues fluctuate from their original position. From the entire MD simulation trajectories, the RMSF of hRRM1 amino acids was calculated and is plotted in Figure 8. It is clearly visible from Figure 8 that with a few exceptions at both terminals, all amino acids of hRRM1 bound with the proposed hRRM1 inhibitors as well as gemcitabine and NSAH were found to fluctuate almost in comparable modus. Average RMSF was found to be 0.220, 0.188, 0.177, 0.177, 0.357, 0.176, and 0.296 nm with binding with gemcitabine, NSAH, BD_1, BD_7, BD_8, BD_10, and BD_14, respectively. The similar fluctuations of hRRM1 amino acids bound with all hRRM1 modulators and lower average values clearly substantiated that each of the amino acids fluctuated in an almost similar fashion and held the small molecules in dynamic states. 

#### 3.3.3. Radius of Gyration

**Figure 9:**
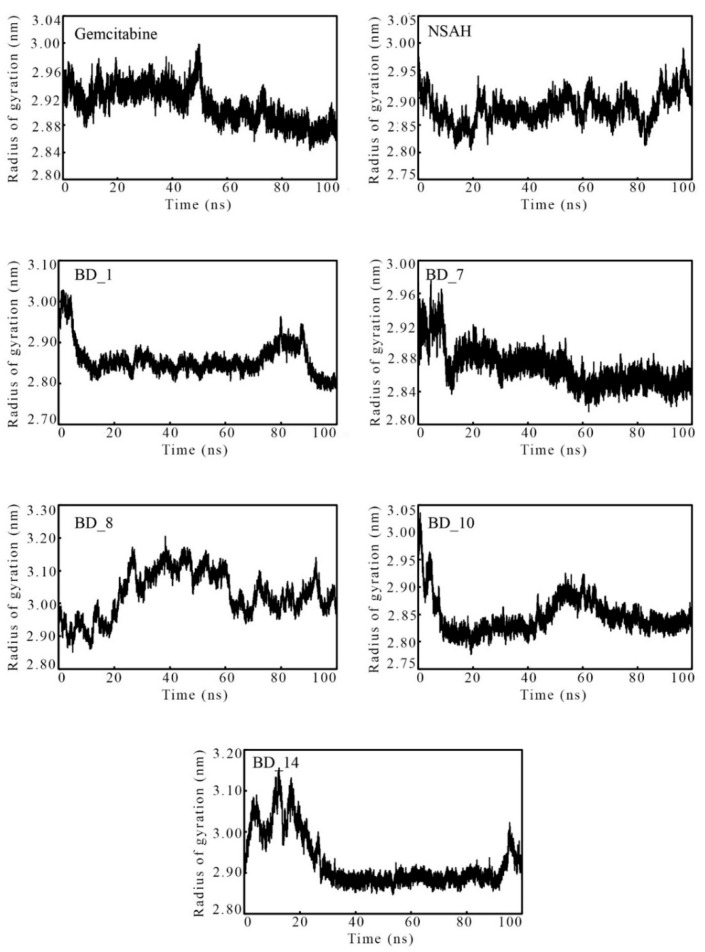
Radius of gyration against the time of the simulation of hRRM1 bound with gemcitabine, NSAH, BD_1, BD_7, BD_8, BD_10, and BD_14.

The compactness and rigidity of any MD simulation system can be assessed using the RoG parameter. A lower RoG value explains folding, whereas a higher value of RoG indicates the unfolding of the protein. The RoG of each frame was calculated, and it is depicted in Figure 9. Not a single frame was found to have an abnormal deviation from its original position. It is important to note that frames among all systems varied between 2.776 and 3.205 nm. Additionally, the average RoG of the systems bound with the proposed hRRM1 inhibitors was found to be much closer to gemcitabine and NSAH. Hence, the above observations clearly were substantiated and explained the steady deviation of the system.

#### 3.3.4. Intermolecular Hydrogen Bonds Analysis during MD Simulation

**Figure 10:**
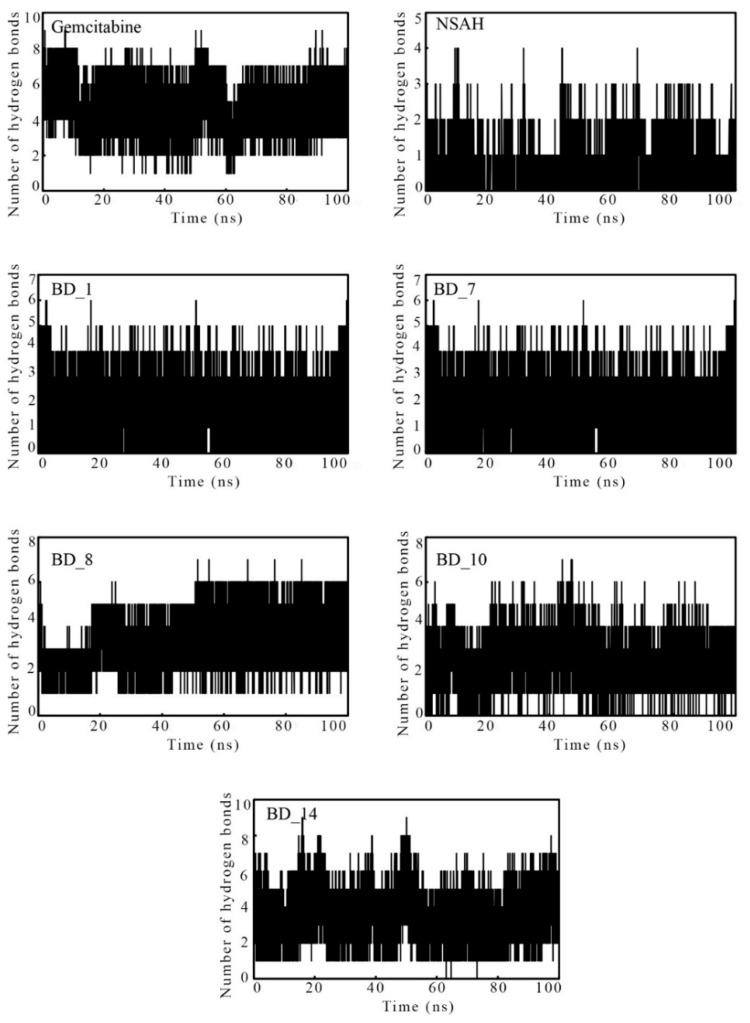
Distribution of hydrogen bond profile of gemcitabine, NSAH, BD_1, BD_7, BD_8, BD_10, and BD_14 towards hRRM1 in the course of MD simulation.

For the stability of the protein and ligand complex, the number of intermolecular hydrogen bonds is one of the crucial factors to keep them together in dynamic states. Due to conformational changes in the MD simulation processing, a number of existing HBs break, and a new set of bonds is formed. The MD simulation trajectories were used to calculate the number of HBs between the hRRM1 active site amino acids and ligands, and it is given in Figure 10. Gemcitabine and BD_8 were found to possess at least one HB in each of the 100,000 frames. Similarly, BD_14 was also found to retain at least one HB except for a very few frames. A much smaller number of HBs was found between hRRM1 and NSAH, although among the proposed hRRM1 molecules, BD_1 and BD_7 were found to have a number of frames without any HBs, but a large number of frames also formed HBs up to six for both molecules. The variation of the number of HBs in different frames in each molecule explained that each molecule went through a conformational ensemble during the course of MD simulation. Therefore, the existence of HBs between hRRM1 and its ligand substantiated and corroborated that the hRRM1 kept the small molecules tightly inside the receptor cavity.

### 3.4. Binding Free Energy Using MM-GBSA Approach and Per-Residue Decomposition Energy

The *ΔG_bind_* values of the proposed five molecules and the two standard molecules, gemcitabine and NSAH, were calculated from 100 ns MD simulation trajectories through the MM-GBSA approach. Calculated *ΔG_bind_* values and standard deviations of gemcitabine, NSAH, BD_1, BD_7, BD_8, BD_10, and BD_14 towards hRRM1 are given in Table 6. Moreover, for the above molecules, binding free energy was also calculated from the 10 ns MD simulation (Table 4) during the screening of the molecule. From both Table 4 and Table 6, it can be seen that the binding affinity of each molecule changed towards hRRM1 on the extension of the MD simulation. In particular, a small *ΔG_bind_* of gemcitabine was reduced; that is, its affinity towards hRRM1 increased. On the other hand, the affinity of NSAH was decreased after 100 ns of MD simulation analysis. In the comparison of *ΔG_bind_* from 10 to 100 ns MD simulation trajectories, it was observed that all proposed molecules enhanced their affinity towards hRRM1. The possible reason behind the increase in affinity might be the more conformational analysis in the longer time span of MD simulation. Overall, the high negative *ΔG_bind_* values of each of the proposed inhibitors undeniably showed that they possess a strong affinity towards hRRM1.

**Figure 11:**
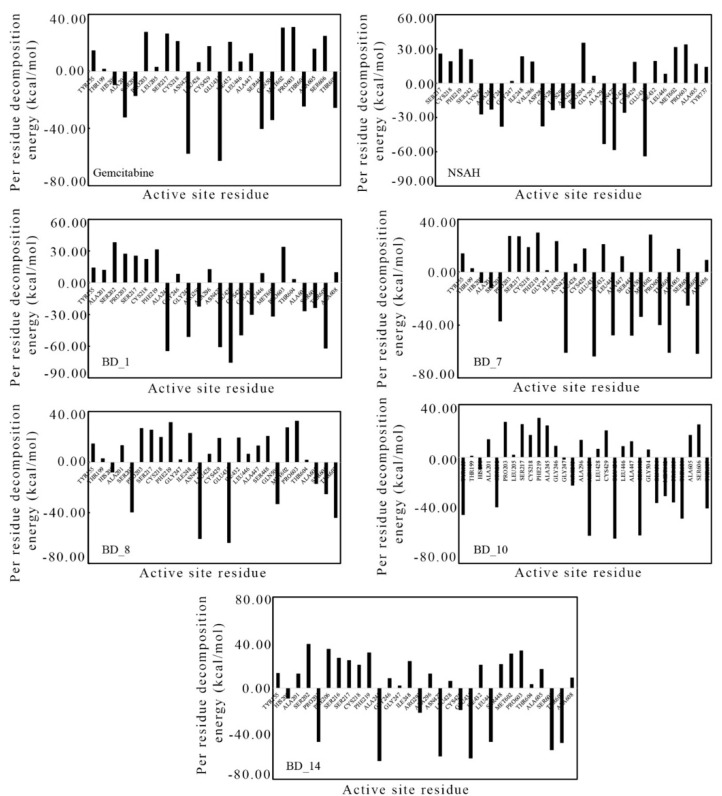
Per-residue decomposition energy of hRRM1 amino acids present around 5 Å of bound ligand.

The amino acids around the bound ligand are the key contributors to the binding interaction, and they can be assessed through the calculation of per-residue decomposition energy. From each MD simulation trajectory, the per-residue decomposition energy of amino acids around 5 Å of bound ligand was calculated and is plotted in Figure 11. The high negative per-residue decomposition energy of any amino acid indicated the strong contribution toward the binding of ligands, and it can be corroborated by the binding interaction analyses in molecular docking. Especially, the per-residue decomposition energy of ligand binding amino acids was checked, and it was found that almost all ligand-binding amino acids for each ligand showed high negative values. The above observation clearly corroborated the binding interaction analysis in the molecular docking study and per-residue decomposition energy from the MD simulation.

### 3.5. Post MD Simulation Binding Interaction Analysis

During and after the MD simulation time, the binding interactions were investigated by extracting the protein–ligand complexes at different time intervals. Particularly, after successful completion of the MD simulation, protein–ligand complexes at the different time frames of 0, 25, 50, 75, and 100 ns were extracted and their binding interactions analyzed using PLIP. The ligand-binding amino acids of each mentioned time frame along with the associated interacting amino acids found in the molecular docking as well as the MD simulation study are given in Table 7. 

**Figure 12:**
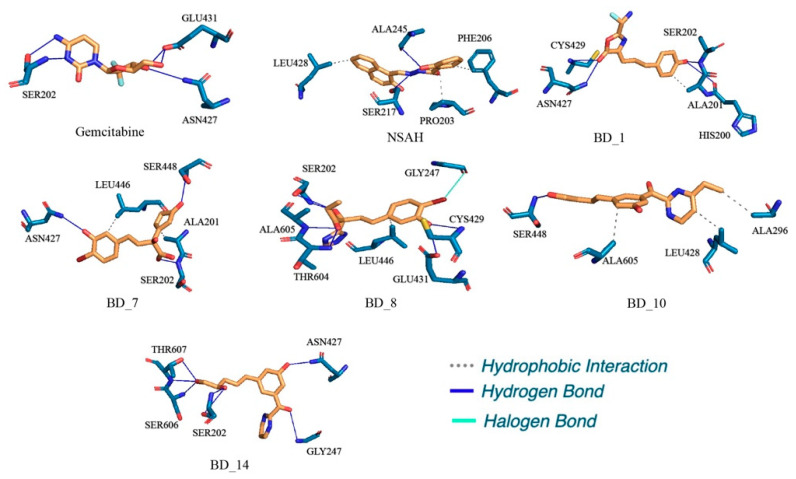
Post-MD simulation binding interactions profile of identified hRRM1 inhibitors–modulators at 100 ns.

Figure 12 shows a representation of binding interactions between hRRM1 and putative ligands at 100 ns. The initial binding position of the ligand and any displacement during the MD simulation (at 100 ns) were investigated and compared using binding interaction profiles of the protein–ligand complexes.

Both standard molecules, gemcitabine and NSAH, were found to retain a number of binding interactions after MD simulation similar to molecular docking. As observed, amino acid residues SER202 and GLU431 were found to interact with gemcitabine in molecular docking, and these residues also retained their HB interaction after 100 ns. Similarly, NSAH was found to interact with LEU428 and ALA245 in both pre- and post-MD simulation complexes. Upon exploring the binding interactions of BD_1 with hRRM1 in molecular docking and post-MD simulation, it was found that ASN427 and CYS429 successfully reserved their interactions. From Table 7 and Figure 12, it can be seen that BD_7 retained a number of binding interactions in pre- and post-MD simulation complexes. Residues ALA201 and LEU446 were seen to form hydrophobic interactions with BD_7 in molecular docking, and the same amino acids retained hydrophobic interactions after 100 ns of MD simulation. It is interesting to note that SER202, ASN427, GLU431, PRO603, and THR607 preserved their HB interactions with BD_7 after the completion of the simulation. One hydrophobic and two HB interactions between BD_8 and hRRM1 remained conserved in molecular docking and post-MD simulation through LEU446, SER202, and GLU431. A halogen bond was newly formed between BD_8 and GLY247. Interestingly this halogen bond was not found in the molecular docking study and might be a change of conformational orientation that brought the halogen atom near GLY427 that favored the binding interaction. Except for SER448, no other hRRM1 amino acids were found to retain their binding interactions with BD_10 after MD simulation. In the case of BD_14, three amino acids, ASN427, SER606, and THR607, were commonly seen to form HB interactions in pre- and post-MD simulations. 

**Figure 13:**
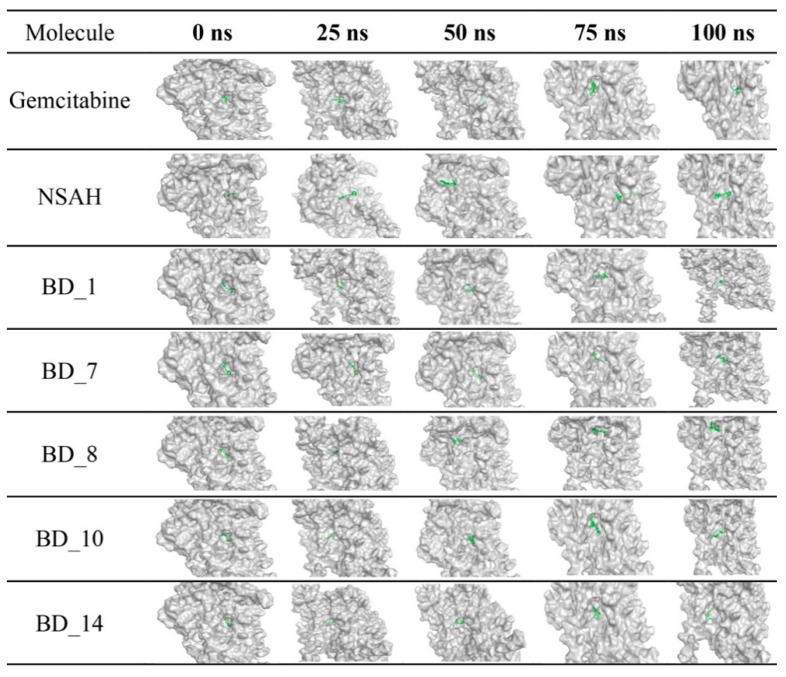
Binding mode in the 3D surface view of hRRM1 inhibitors in 0, 25, 50, 75, and 100 ns of MD simulation.

Beyond the above common binding interactions between hRRM1 and the proposed molecules, a number of new bonds were seen to form, and also a few existing bonds were lost during the MD simulation. It is also important to note that a number of hydrophobic and HB interactions remained intact after 25, 50, and 75 ns of MD simulation. Therefore, from the post-MD simulation-based analysis, it is quite clear that during the MD simulation, although some sort of small conformational changes were observed for hRRM1, each ligand remained at the active site for a longer time span. The dynamicity of the small molecules was also substantiated through the loss of existing contacts and the formation of a new set of binding interactions. The position and binding mode of each ligand at 0, 25, 50, 75, and 100 ns were represented in the ‘stick’ view mode, as is given in Figure 13. It can be seen that all molecules changed their conformational orientation slightly, but not moved away from the active site. The above observations certainly indicated that all molecules remained inside the active site throughout the simulation and definitely possessed a strong binding affinity towards hRRM1.

## 4. Conclusions

A set of novel hRRM1 inhibitors was generated through the de novo design approach for the effective therapeutic application in BTC. A number of advanced computational drug discovery approaches such as molecular docking, *k*-means pharmacophoric clustering, NIB screening, pharmacokinetics, and toxicity assessments was carried out to wipe out inactive molecules. Following the above, a total of 17 molecules was found to be crucial for hRRM1 activity modulation. Further, the potentiality of the above molecules was confirmed through MD simulation-based binding free energy calculation, PLANTS-based binding energy, *PharmFrac*, and absolute binding free energy. Upon detailed analysis, the top five molecules from the above set of 17 were further considered for binding interactions analysis and extension of MD simulation. Several potential binding interactions between the proposed molecules and hRRM1 revealed the strong affection of the molecules. A number of common binding interactions with gemcitabine and NSAH was also favored, being promising hRRM1 inhibitors. From MD simulation trajectories, several statistical parameters explained the compactness and stability between protein and the proposed molecules in dynamic states. High negative MM-GBSA-based binding free energy showed a strong affinity of the molecules toward hRRM1. Post-MD simulation frames also revealed a number of common binding profiles with molecular docking. Hence, the final proposed molecules could emerge as promising hRRM1 inhibitors for the management of BTC, subject to experimental validation.

## Figures and Tables

**Table 1:** Experimental inhibitory concentration and ShaEP score based on three models for known hRRM1 inhibitors.

Molecules	Log (*IC*_50_) in µM	Model I	Model II	Model III
Gemcitabine	2.622	0.451	0.513	0.645
M40128	2.396	0.530	0.529	0.454
M777989	2.879	0.307	0.720	0.548
M951562	2.807	0.489	0.681	0.568
NSAH	2.495	0.389	0.524	0.495
M859980	3.420	0.654	0.653	0.597
**Correlation coefficient (*R*)**		**0.480**	**0.660**	**0.535**

**Table 2:** PLANTS-based docking score and absolute binding affinity of identified hRRM1 inhibitors/modulators molecules.

Mols	PLANTS Score (kcal/mol)	SwissDock ΔG (kcal/mol)	K_DEEP_ ΔG (kcal/mol)
Gemcitabine	−67.106	−7.46	−5.819
NSAH	−80.290	−7.19	−5.520
BD_1	−83.636	−7.70	−6.961
BD_2	−76.806	−7.52	−8.999
BD_3	−75.577	−7.70	−5.986
BD_4	−73.217	−7.22	−7.777
BD_5	−75.217	−7.42	−8.313
BD_6	−89.560	−8.56	−8.092
BD_7	−92.362	−8.16	−8.854
BD_8	−82.278	−7.92	−8.612
BD_9	−83.276	−7.81	−8.109
BD_10	−79.143	−7.74	−7.821
BD_11	−71.442	−7.96	−7.512
BD_12	−87.175	−7.97	−9.222
BD_13	−61.565	−7.50	−7.382
BD_14	−84.934	−8.41	−7.711
BD_15	−78.741	−7.65	−8.140
BD_16	−72.471	−7.59	−9.358
BD_17	−83.795	−8.34	−8.129

**Table 3:** *cPharmFrac* and *PharmFrac* of hRRM1 inhibitors obtained from Python RDKit.

Molecules	^1^ HBD	^2^ HBA	^3^ HY	^4^ RA
	***cPharmFrac***			
Gemcitabine +	0.273	0.364	0.182	0.182
NSAH				
	***PharmFrac***			
BD_1	0.182	0.364	0.364	0.091
BD_2	0.200	0.300	0.300	0.200
BD_3	0.300	0.200	0.400	0.100
BD_4	0.214	0.357	0.286	0.143
BD_5	0.133	0.467	0.267	0.133
BD_6	0.167	0.417	0.333	0.083
BD_7	0.214	0.357	0.357	0.071
BD_8	0.143	0.357	0.357	0.143
BD_9	0.286	0.286	0.357	0.071
BD_10	0.231	0.231	0.462	0.077
BD_11	0.231	0.308	0.385	0.077
BD_12	0.267	0.200	0.400	0.133
BD_13	0.231	0.462	0.231	0.077
BD_14	0.286	0.286	0.357	0.071
BD_15	0.214	0.286	0.357	0.143
BD_16	0.200	0.333	0.333	0.133
BD_17	0.231	0.308	0.385	0.077

^1^ HB donor; ^2^ HB acceptor; ^3^ hydrophobic; ^4^ ring aromatic

**Table 4:** Binding energy of the 17 best hRRM1 inhibitors, gemcitabine, and NSAH through the MM-GBSA approach.

Molecule	*ΔG_bind_* kcal/mol	Standard Deviation
Gemcitabine	−36.65	3.15
NSAH	−19.53	2.91
BD_1	−24.62	3.74
BD_2	−23.75	2.81
BD_3	−17.79	4.11
BD_4	−12.59	3.94
BD_5	−12.45	3.84
BD_6	−20.74	5.22
BD_7	−48.27	4.68
BD_8	−39.72	3.41
BD_9	−18.27	4.12
BD_10	−27.02	3.15
BD_11	−14.56	5.82
BD_12	−15.04	4.91
BD_13	−17.89	5.01
BD_14	−29.75	4.75
BD_15	−9.74	6.16
BD_16	−14.65	3.14
BD_17	−7.08	4.22

**Table 5:** Statistical parameters from MD simulation trajectories for hRRM1 inhibitors.

Parameters		Gemcitabine	NSAH	BD_1	BD_7	BD_8	BD_10	BD_14
Backbone RMSD (nm)	Min.	0.001	0.001	0.001	0.000	0.001	0.000	0.000
	Max	1.263	0.681	0.947	1.106	1.599	0.875	1.250
	Avg	1.057	0.442	0.806	0.884	1.092	0.785	0.611
Ligand RMSD (nm)	Min	0.000	0.000	0.000	0.000	0.000	0.000	0.000
	Max	0.114	0.187	0.214	0.109	0.165	0.313	0.209
	Avg	0.044	0.072	0.107	0.053	0.099	0.199	0.123
RMSF (nm)	Min	0.063	0.052	0.051	0.051	0.071	0.053	0.066
	Max	1.525	2.286	1.731	1.242	3.803	1.988	2.210
	Avg	0.220	0.188	0.177	0.177	0.357	0.176	0.296
RoG (nm)	Min	2.843	2.804	2.780	2.815	2.851	2.776	2.846
	Max	2.999	2.991	3.028	2.976	3.205	3.035	3.156
	Avg	2.911	2.881	2.858	2.870	3.029	2.846	2.926

Min: minimum; Max: maximum; Avg: average.

**Table 6:** Binding free energy of final five BTC molecules calculated from 100 ns MD simulation trajectory.

Molecule	*ΔG_bind_* kcal/mol (Std. Dev.)
Gemcitabine	−39.13 (±3.07)
NSAH	−17.53 (±3.41)
BD_1	−31.13 (±3.52)
BD_7	−55.27 (±4.75)
BD_8	−47.11 (±2.99)
BD_10	−35.31 (±3.02)
BD_14	−37.39 (±4.65)

Std. Dev.: standard deviation.

**Table 7:** Post-MD simulation binding interaction profile at 0, 25, 50, 75, and 100 ns.

		Binding Interaction Analysis					
Molecule	Bonds	Mol. Dock.	Post-MD Simulation				
			0 ns	25 ns	50 ns	75 ns	100 ns
Gemcitabine	HY	ALA201, THR604	-	-	-	-	-
	HB	HIS200, SER202, GLU431, SER448, THR607	SER202, SER217, GLU431, SER448, THR607	SER202, GLU431, SER448	SER202, SER448, THR607	ASN427, GLU431, ALA605, SER606, THR607	SER202, ASN427, GLU431
NSAH	HY	GLN288, LEU428	ALA245, GLN288, LEU428	PHE206	ARG153	PRO203, GLN214, ALA245, ALA296	PRO203, PHE206, LEU428
	HB	ALA245, GLN246, ALA296	ALA245, ARG293, ARG293, ALA296	SER202	SER154	-	SER217, ALA245
BD_1	HY	LEU446, MET602, ALA605	-	-	-	ALA201, LEU446	-
	HB	ALA245, GLY247, ASN427, LEU428, CYS429, SER606, THR607	ALA245, ASN427, CYS429, SER606, THR607	SER202, ASN427, LEU428	SER202, SER217, ASN427, LEU428, THR607	SER202, SER217, GLY247, ASN427, LEU428, CYS429, THR607	HIS200, SER202, ASN427, CYS429
BD_7	HY	ALA201, LEU446, LEU446, THR604, THR607	ALA201, LEU446	ALA201, LEU446	ALA201, LEU446	ALA201	ALA201, LEU446
	HB	SER202, ASN427, GLU431, SER448, PRO603, SER606, THR607	SER202, ASN427, GLU431, PRO603, SER606	SER202, ASN427, GLU431, PRO603, THR607	SER202, ASN427, GLU431, PRO603, THR607	SER202, ASN427, GLU431, PRO603, THR607	SER202, ASN427, GLU431, PRO603, THR607
BD_8	HY	LEU446, ALA605	LEU446	LEU446, MET602	LEU446, ALA605	LEU446, MET602	LEU446
	HB	SER202, ASN427, GLU431, SER606, THR607	SER202, ASN427, GLU431, SER606, THR607	SER202, ASN427, ALA605	SER202, ASN427, ALA605	SER202, CYS429, GLU431, ALA447, SER448, THR604, ALA605	SER202, CYS429, GLU431, THR604, ALA605
	HAL		GLY247	GLY247	GLY247	GLY247	GLY247
BD_10	HY	ALA201, MET602, PRO603, THR604	ALA201	-	LEU428, THR607	ALA201, LEU428	ALA296, LEU428, ALA605
	HB	TYR155, SER202, GLY247, SER448, THR607	TYR155, SER202, ALA245, ARG293, SER448, SER606, THR607	TYR155, SER202, ARG293, SER448, SER606, THR607	TYR155, SER202, ARG293, ARG293, SER448, THR607	SER202, SER217, SER448, THR607	SER448
	pi-Cation	-	-	-	ARG293	-	-
BD_14	HY	PRO203, LEU446	ALA428	-	PRO203	PRO203	-
	HB	ALA245, ASN427, LEU428, CYS429, SER606, THR607	SER202, ASN427, SER606, THR607	SER202, SER217, GLY247, SER606, THR607	SER202, GLY247, THR607	SER202, GLY247, ASN427, SER606	SER202, GLY247, ASN427, SER606, THR607

Mol. Dock: molecular docking; HY: hydrophobic bond; HB: hydrogen bond; HAL: halogen bond.

## Data Availability

Not applicable.

## Supplementary Materials

The following supporting information can be downloaded at: https://www.mdpi.com/article/10.3390/biom12091279/s1, Figure S1: Superimposed structure of co-crystal and best docked pose of GDP. The RMSD was calculated 1.235 Å; Figure S2: hRRM1 backbone RMSD bound with proposed molecules and gemcitabine and NSAH; Figure S3: RMSD of hRRM1 proposed molecules and gemcitabine and NSAH; Figure S4: The radius of gyration of hRRM1 bound with proposed molecules and gemcitabine and NSAH; Figure S5: Intermolecular hydrogen bonds between hRRM1 and proposed molecules and gemcitabine and NSAH; Table S1: Number of pharmacophoric features in hRRM1 molecules calculated using Python RDKit.
