# Supplementary material for: Identification of Novel Ribonucleotide Reductase Inhibitors for Therapeutic Application in Bile Tract Cancer: An Advanced Pharmacoinformatics Study
Source: Biomolecules. 2022 Sep 10;12(9):1279. doi: 10.3390/biom12091279 (PMC9496582; doi:10.3390/biom12091279)
Supplement: Supplementary file 1 [file biomolecules-12-01279-s001.zip › biomolecules-1895084-supplementary.pdf]

Identification of novel ribonucleotide reductase inhibitors for therapeutic application in Bile tract cancer: An advanced pharmacoinformatics study

Md Ataul Islam, Mayuri Makarand Barshetty, Sridhar Srinivasan, Dawood Babu Dudekula, V. P. Subramanyam Rallaband, Sameer Mohammed, Sathishkumar Natarajan, Junhyung Park

Supplementary information

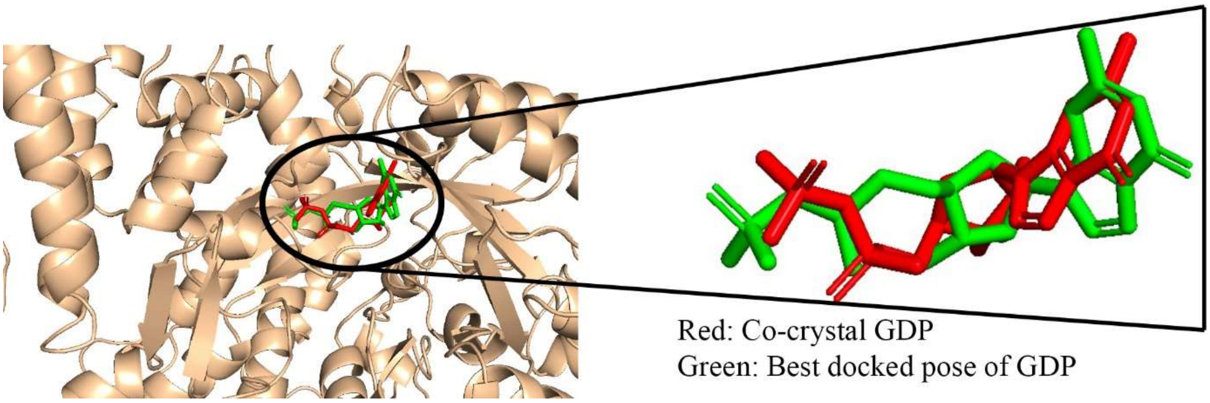

Figure S1. Superimposed structure of co-crystal and best docked pose of GDP. The RMSD was calculated 1.235 Å

Table S1. Number of pharmacophoric features in hRRM1 molecules calculated using Python RDKit

Molecules	[1]HBD	[2]HBA	[3]HY	[4]RA
Gemcitabine	3	5	0	1
NSAH	3	3	4	3
BD_1	2	4	4	1
BD_2	2	3	3	2
BD_3	3	2	4	1
BD_4	3	5	4	2
BD_5	2	7	4	2
BD_6	2	5	4	1
BD_7	3	5	5	1
BD_8	2	5	5	2
BD_9	4	4	5	1
BD_10	3	3	6	1
BD_11	3	4	5	1

BD_12	4	3	6	2
BD_13	3	6	3	1
BD_14	4	4	5	1
BD_15	3	4	5	2
BD_16	3	5	5	2
BD_17	3	4	5	1

[1]HB donor; [2]HB acceptor; [3]Hydrophobic; [4]Ring aromatic

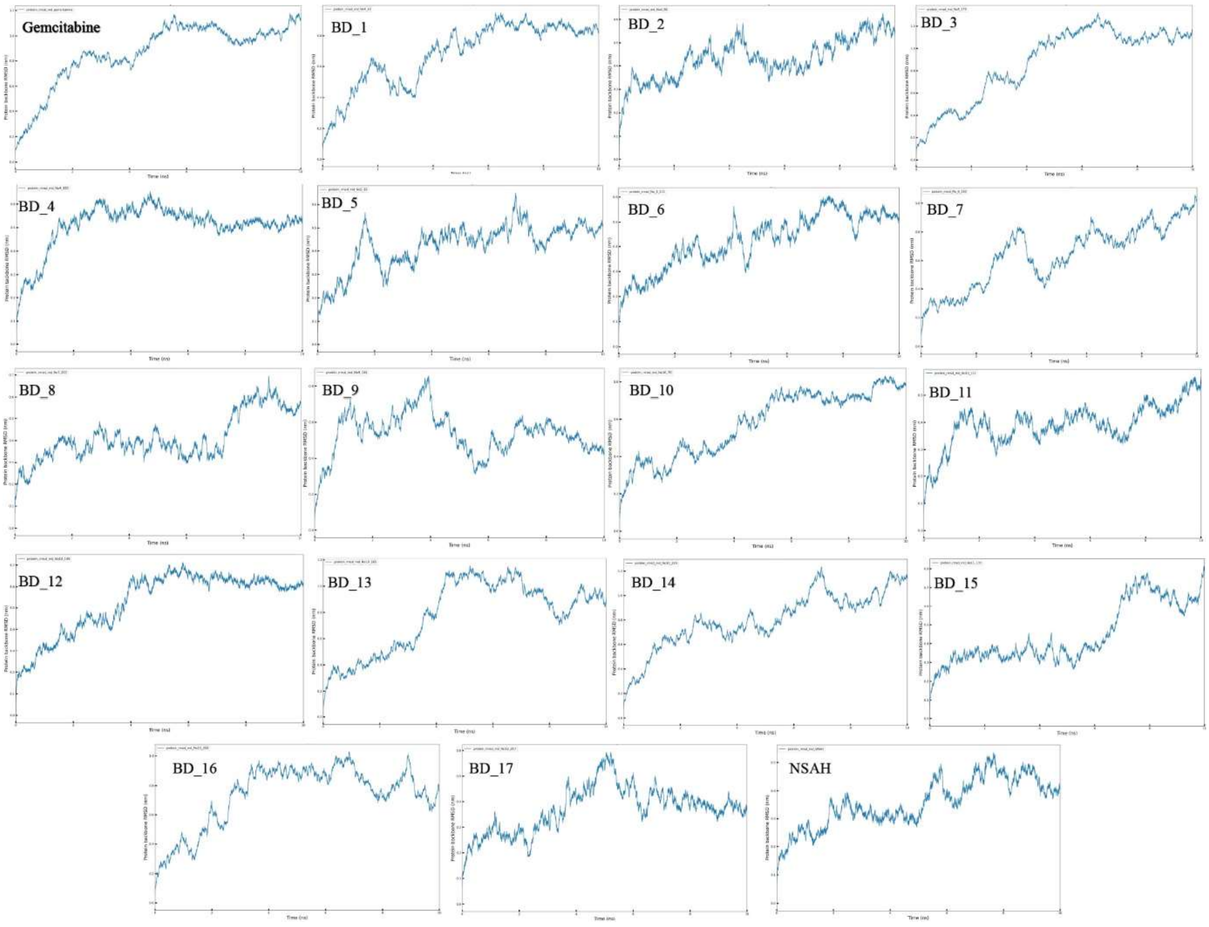

Figure S2. hRRM1 backbone RMSD bound with proposed molecules, and, Gemcitabine and NSAH

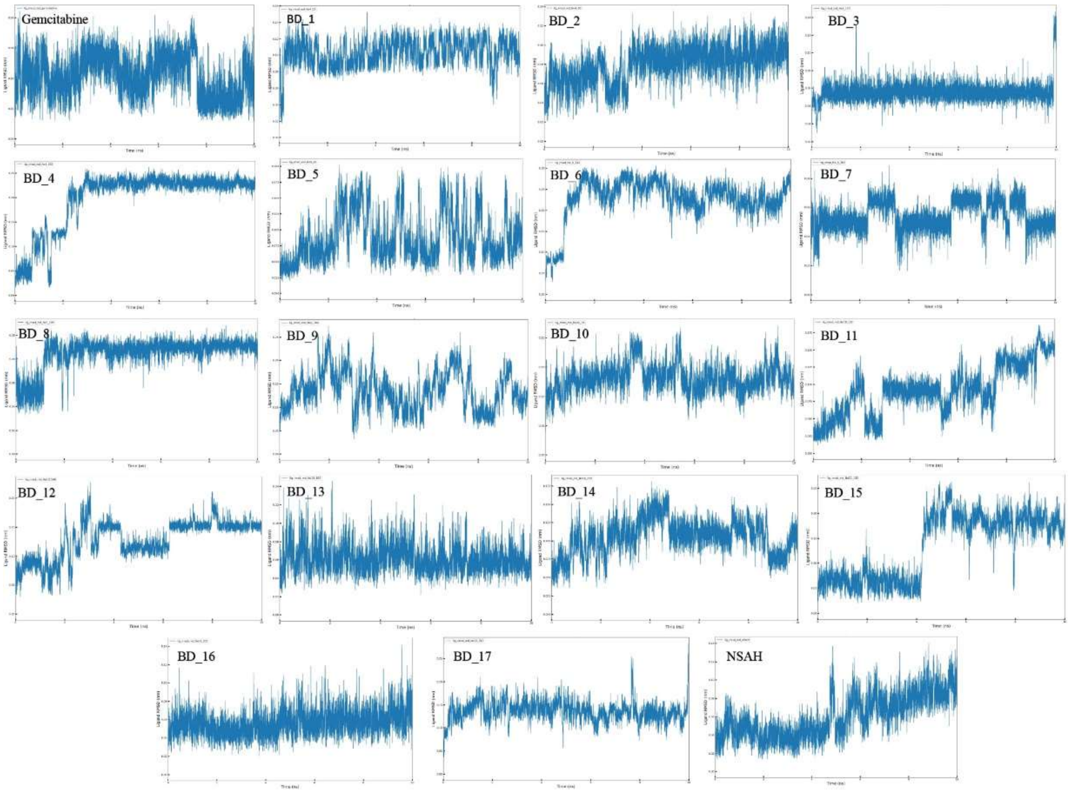

Figure S3. RMSD of hRRM1 proposed molecules, and, Gemcitabine and NSAH

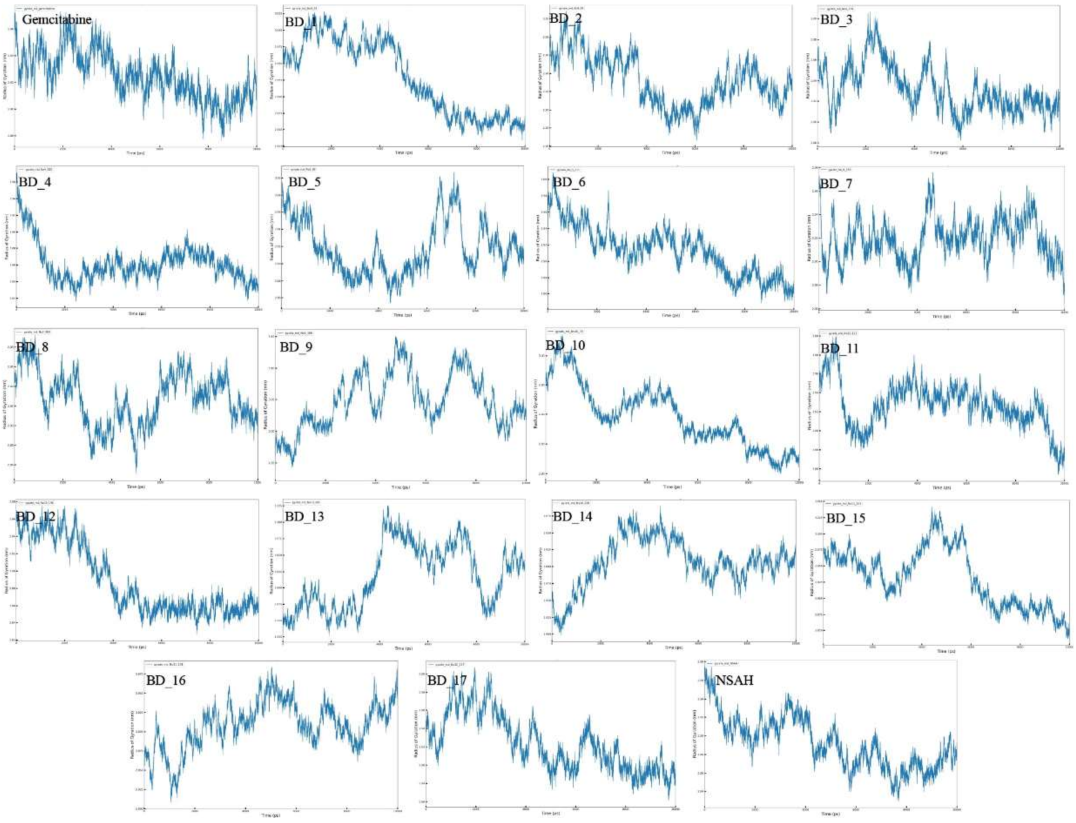

Figure S4. The radius of gyration of hRRM1 bound with proposed molecules, and, Gemcitabinbe and NSAH

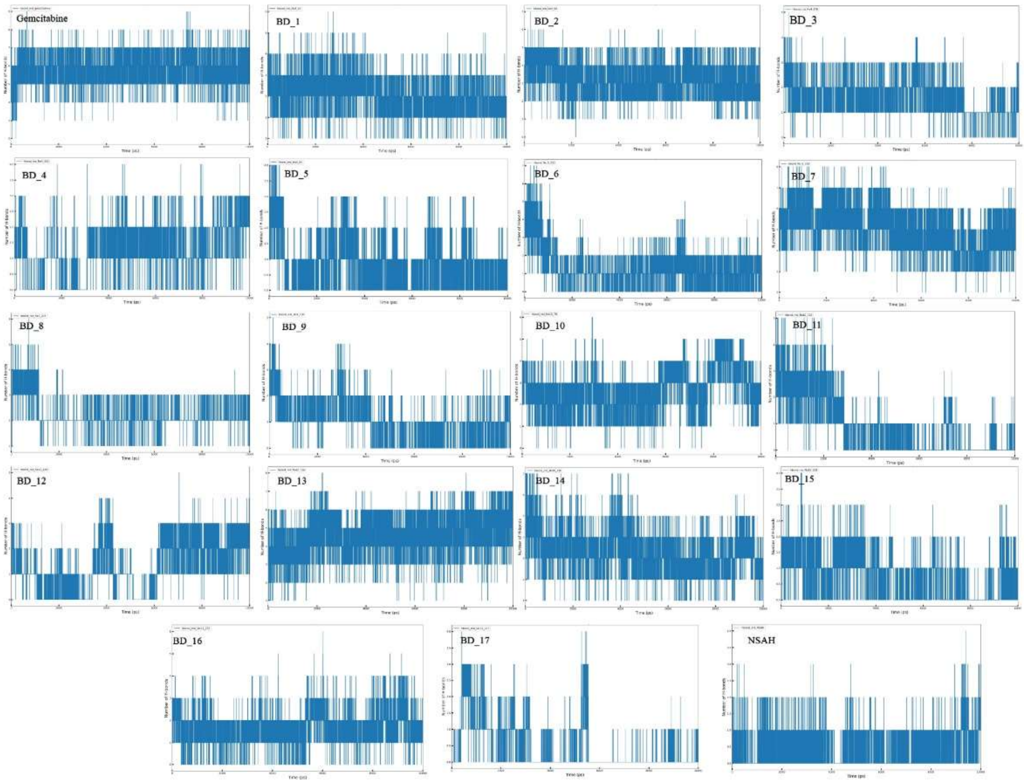

Figure S5. Intermolecular hydrogen bonds between hRRM1 and proposed molecules, and, Gemcitabine and NSAH
